# Transcriptome Analysis of Arcobacter butzleri Infection in a Mucus-Producing Human Intestinal *In Vitro* Model

**DOI:** 10.1128/spectrum.02071-22

**Published:** 2023-01-09

**Authors:** Davide Buzzanca, Valentina Alessandria, Cristian Botta, Negin Seif Zadeh, Ilario Ferrocino, Kurt Houf, Luca Cocolin, Kalliopi Rantsiou

**Affiliations:** a Department of Agricultural, Forest and Food Sciences, University of Turin, Turin, Italy; b Department of Veterinary and Biosciences, Faculty of Veterinary Medicine, Ghent University, Merelbeke, Belgium; Iowa State University

**Keywords:** *Arcobacter butzleri*, *Arcobacteraceae*, RNA-seq, transcriptome, virulence genes

## Abstract

Arcobacter butzleri is a foodborne pathogen belonging to the *Arcobacteraceae* family. This Gram-negative bacterium is found in water, food, and various organisms, including farm animals, clams, and fish. Moreover, A. butzleri has been isolated from human stool samples, where it was associated with gastrointestinal symptoms such as diarrhea. The present study focused on the transcriptome analysis of three A. butzleri strains isolated from human stools and displaying variable virulence potential *in vitro*. We used a mucus-producing human intestinal *in vitro* model (Caco-2/HT29-MTX-E12) to study the colonization and invasion abilities of the three A. butzleri strains. The ability of all three A. butzleri strains to colonize our *in vitro* model system was subsequently confirmed. Moreover, transcriptomics showed the upregulation of putative virulence genes. Among these genes, *tonB*, *exbB*, and *exbD*, which belong to the same operon, were upregulated in strain LMG 11119, which also had the greatest colonization ability. Moreover, genes not currently considered A. butzleri virulence genes were differentially expressed during cell model colonization. The main functions of these genes were linked to organic acid metabolism and iron transport and particularly to the function of the TonB complex.

**IMPORTANCE** Recent advancements in the genomic characterization of A. butzleri revealed putative virulence genes and highlighted the possible pathogenic mechanisms used by this foodborne pathogen. It is therefore possible to study the transcriptomes of these bacteria to explore possible virulence mechanisms under conditions that mimic the infection process. The transcriptome and colonization/invasion analyses that we performed in this study enabled the evaluation of A. butzleri-mediated infection of the mucus-producing human intestinal *in vitro* model. We confirmed the upregulation of previously proposed virulence genes in the A. butzleri strains. In addition, we identified the differential expression of a number of other genes, which are not currently thought to be associated with virulence, in three A. butzleri strains during infection of mucus-producing human epithelial cells. Changes in the concentration of acetic acid and the upregulation of genes associated with organic acid metabolism during host-pathogen contact were also observed. These findings highlight the importance of previously unreported genes in the virulence mechanisms of A. butzleri.

## INTRODUCTION

Arcobacter butzleri (recently proposed as Aliarcobacter butzleri) is a Gram-negative bacterium belonging to the *Arcobacteraceae* family (1). A. butzleri can be isolated from human stools or from foods such as meat, dairy, and vegetables. For this reason, A. butzleri is considered a foodborne pathogen (1, 2). In humans, A. butzleri infection has been associated with various gastrointestinal symptoms, such as diarrhea and abdominal pains (2, 3). A. butzleri has also been isolated from animals such as clams, chickens, pigs, cattle, dogs, and cats (3). Animals infected with A. butzleri are typically asymptomatic; thus, the prevalence of this bacterium in farms is underestimated (3). The increase in A. butzleri antibiotic resistance and the reported human clinical cases linked to A. butzleri infections highlight the need to expand our limited knowledge of this microorganism (2, 3).

In recent years, whole-genome sequencing studies have enabled the exploration and functional annotation of genetic sequences associated with A. butzleri virulence and antibiotic resistance (4–6). Ten well-characterized putative A. butzleri virulence genes (*cadF*, *ciaB*, *cj1349*, *hecA*, *hecB*, *mviN*, *pldA*, *irgA*, *tlyA*, and *iroE*) are frequently detected in infected subjects (2, 7). Further characterization of these genes and the discovery of new ones will help identify virulence-associated genetic sequences and improve our understanding of the mechanism of A. butzleri infection. Moreover, the characterization of virulence-related sequences will enable the design of new molecular detection methods for A. butzleri (2, 7, 8). Recently, comparative genomics studies of A. butzleri enabled the identification of genes with different functions, which were directly or indirectly linked to virulence (5, 6). Genetic sequences implicated in the regulation of gene expression, flagellum functionality, chemotaxis, urease activity, and iron transport have been highlighted as putative virulence genes (5, 6). Some of these genes are characterized by sequence hypervariability between strains. This is particularly evident for the gene encoding the PorA protein (5) or genes implicated in lipopolysaccharide synthesis and O-antigen production (6).

Genomic analyses, coupled with physiological observation, can lead to the elucidation of putative virulence-related mechanisms. This can be achieved using *in vitro* cell models, which are an important tool for studying bacterial adhesion and invasion (9). *In vitro* human epithelial models are often used to study the pathogenic mechanisms of intestinal pathogens. For instance, human colon adenocarcinoma cell lines such as Caco-2 (which can differentiate into enterocyte-like cells) (9) and HT29-MTX-E12 (which is known for its mucus-producing ability) (10) are used in mono- or cocultures to mimic the process of bacterial infection (11). Such *in vitro* studies have shown that A. butzleri adheres to and invades host cells by interacting with the mucus produced by the cell line HT29-MTX-E12 (6). Furthermore, tests of colonization or invasion yield biological samples which can be used for transcriptome analysis (12).

However, despite the wealth of genomic and physiological data on A. butzleri, its virulence mechanism is still unclear, and although the annotation of A. butzleri genomes has enabled the identification of putative virulence genes, the gene expression profiles of A. butzleri during the process of infection need to be further investigated (5, 6). Thus, the present study aimed to evaluate the transcriptome of A. butzleri in a human intestinal mucus-producing *in vitro* model, which mimics the *in vivo* infection process.

## RESULTS AND DISCUSSION

### Colonization and invasion ability of A. butzleri and analysis of its transcriptome.

A. butzleri strains LMG 11119, LMG 10828^T^, and 31 were isolated from human clinical samples originating from different geographical areas (Italy, the United States, and Belgium, respectively). These strains were selected because of differences in their genomic (see Fig. S1 in the supplemental material) and virulence features, which were previously described (6). Previously, the higher adhesion of A. butzleri to mucus-producing mixed-cell models (i.e., Caco-2 and HT29-MTX-E12 cells combined in a 9:1 ratio) was observed, compared with the colonization of the Caco-2 (a cell line that lacks mucus production) monoculture model or a mixed Caco-2/HT29 (9:1 ratio) model (6). The higher colonizing ability of A. butzleri in the presence of mucus is in agreement with observations of other Gram-negative pathogens, including Campylobacter jejuni and Helicobacter pylori, which can interact with intestinal mucosal glycoproteins called mucins (13–16).

**FIG 1:**
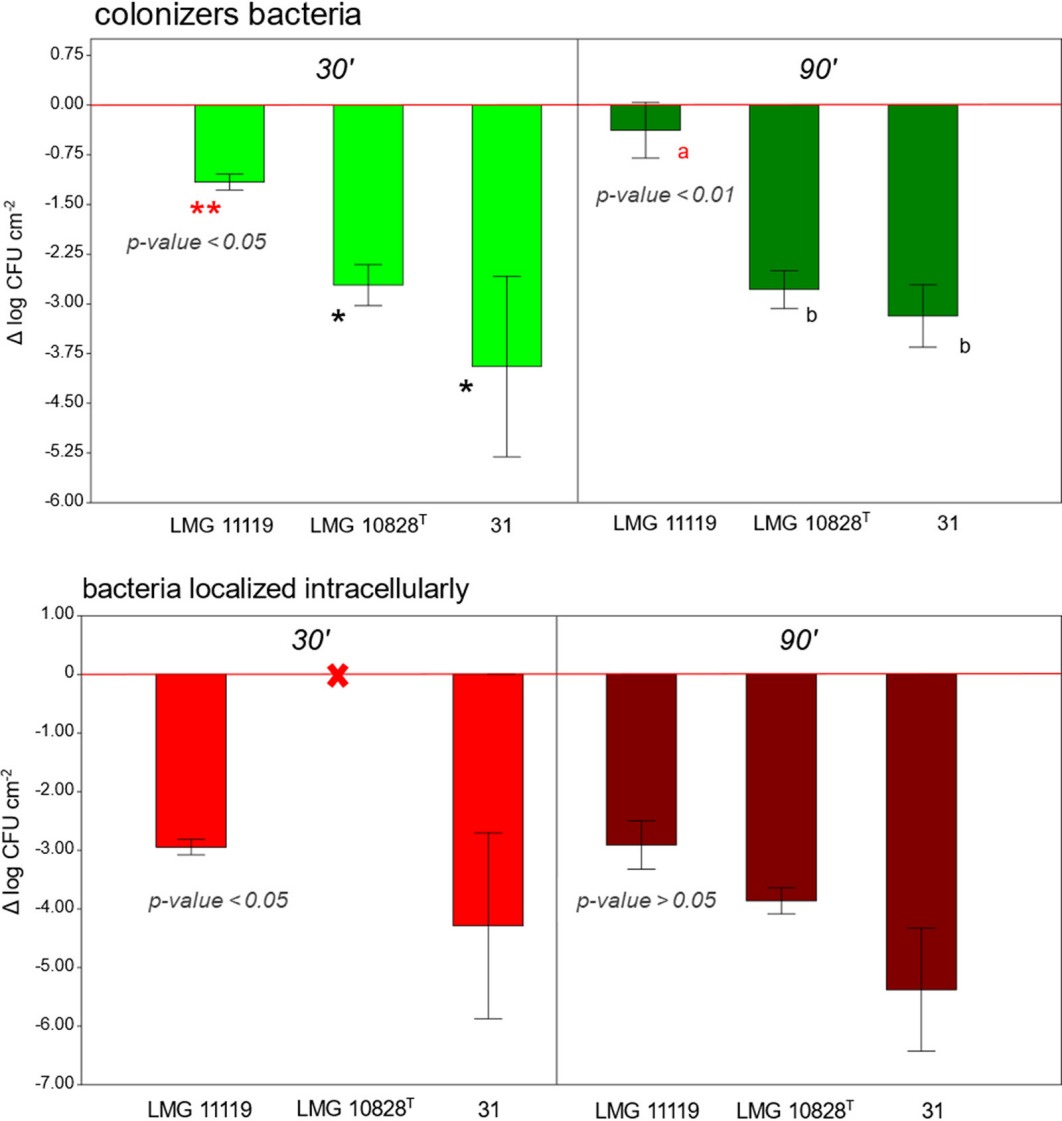
Results of the *in vitro* colonization-invasion assay. The bar chart shows the presence of colonizing (i.e., bacteria that had adhered to or were internalized into host cells) and invading (i.e., bacteria that were internalized into host cells) bacteria after 30 (30’) or 90 (90’) min of bacterium-cell contact. The values for Δlog CFU per square centimeter correspond to adhered and internalized bacteria (see “*In vitro* colonization-invasion assay” for the calculation method). The error bars represent the standard errors of the means (SEM), while the red lines indicate a Δlog of 0 (i.e., if the bacterial load detected is equal to the initial bacterial load used to inoculate the host cell). The red “X” indicates that no bacteria were detected, meaning that no bacterial adhesion or invasion took place. *P* values indicate the statistical significance of differences between strains. One or two asterisks or different letters indicate statistically significant difference in colonization values.

In this study, we used an *in vitro* mucus-producing cell model (Caco-2/HT29-MTX-E12) to evaluate the ability of A. butzleri to colonize and invade the epithelial cell layer after 30 or 90 min of bacterium-host cell contact. All A. butzleri strains were able to colonize the cell layer at both time points of infection; however, strain LMG 11119 was the highest colonizer after both 30 min (*P* value < 0.05) and 90 min (*P* value < 0.01) (Table S1). Regarding invasion, no internalization of LMG 10828^T^ bacteria was observed after 30 min of infection. However, after 90 min, all three strains were detected inside host cells, which confirms that these strains had the ability to invade human cell lines. Although strain LMG 11119 showed a tendency toward greater invasion ability, this was not statistically significant (*P* value > 0.05) (Fig. 1 and Table S1). These data highlight differences in the behavior of the three A. butzleri strains tested, which could also be relevant to their virulence potential. Overall, our findings were in agreement with previously published work documenting the ability of A. butzleri strains to colonize mucus-producing human intestinal cell models *in vitro* (6).

We surmised that the differences in the colonization and internalization behavior of the three A. butzleri strains could be related to differences in gene expression. Therefore, we performed a transcriptome analysis of each strain after the strains had been in contact with the human mucus-producing *in vitro* model for 30 or 90 min. As a control, gene expression in each A. butzleri strain was evaluated after growth in *Arcobacter* agar media or in Dulbecco’s modified Eagle medium (DMEM) after 2 h of adaptation by incubation at 37°C (5% CO_2_). In this way, changes in gene expression not directly linked to contact with the mucus-producing human cell layer could be detected. Furthermore, the DMEM adaptation step was used as a control condition when evaluating the presence of differentially expressed genes (DEGs) after contact with the human host cells.

### Analysis of A. butzleri DEGs upon switching from *Arcobacter* medium to DMEM.

**FIG 2:**
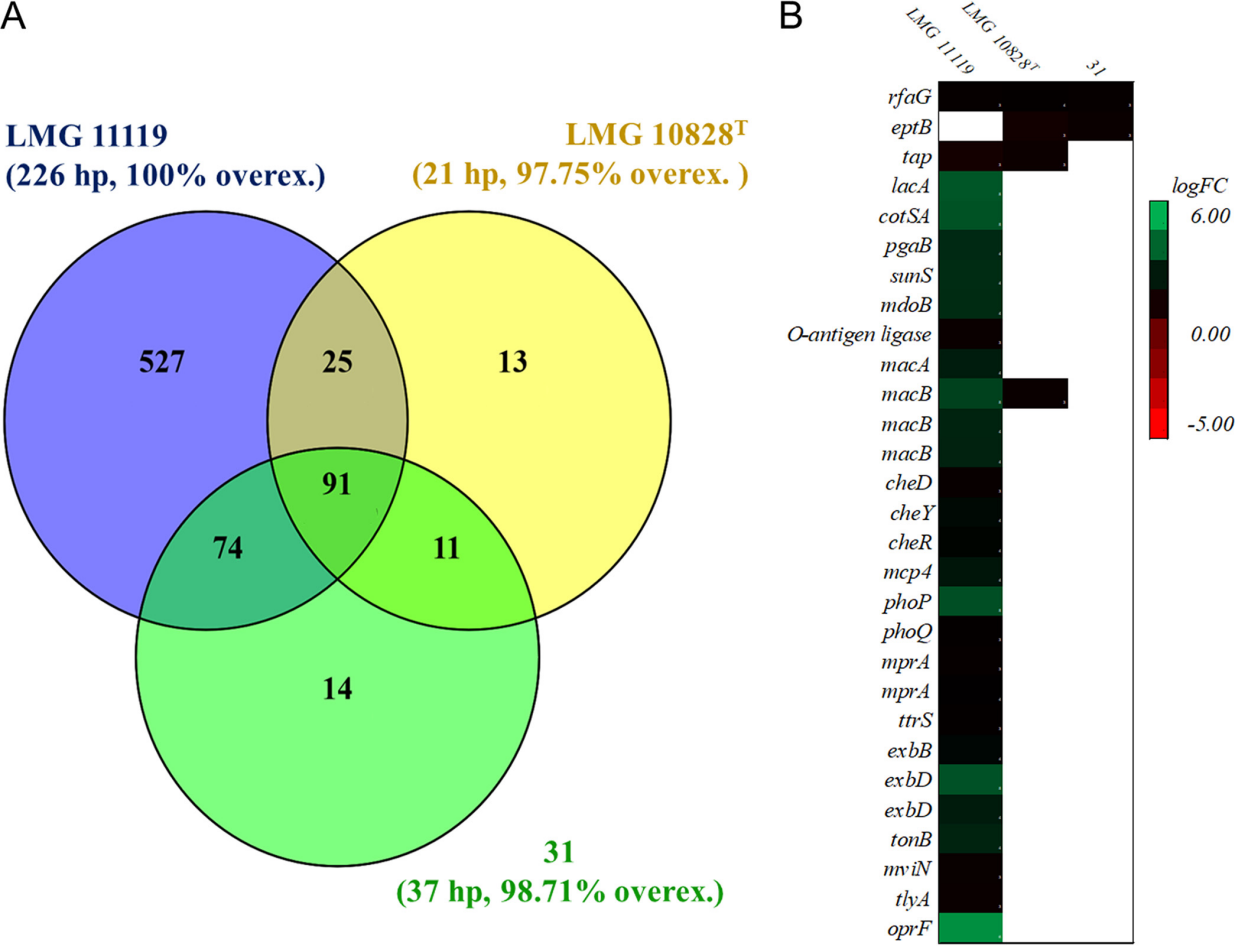
Venn diagram of DEGs in the three of A. butzleri strains (A) and heat map of virulence-associated DEGs after A. butzleri transfer to DMEM (B). The DEGs shown were derived from the comparison of bacteria in DMEM (test) and *Arcobacter* agar (control). The Venn diagram shows the presence of DEGs shared between different A. butzleri strains and present in each strain (hypothetical proteins [hp] excluded). The percentages of upregulated genes (calculated using the total number of DEGs, including hypothetical proteins) are indicated in parentheses below the name of each strain. The A. butzleri DEGs after medium adaptation (via 2 h of incubation in DMEM) are shown in the heat map, alongside potentially virulent DEGs.

The A. butzleri transcriptome was analyzed after 2 h of incubation in DMEM at 37°C; this was the adaptation phase performed before the *in vitro* cell model assay. We observed an increase in gene expression on switching from *Arcobacter* media to DMEM. Differences in the number of DEGs (log fold changes [FC] of >3 and <3; *P* value < 0.05; false-discovery rate [FDR] < 0.05) were observed between strains. More specifically, in strain LMG 11119, 44.69% of the total annotated genes were DEGs, which were all upregulated; in strain LMG 10828^T^, 7.68% of all genes were DEGs, of which 97.75% were upregulated; and in strain 31, 10.77% of all genes were DEGs, of which 98.71% were upregulated (Fig. 2A). According to the Clusters of Orthologous Genes (COG), the DEGs belonged to different gene classes, most of which were involved in signaling pathways associated with translation, ribosomal structure and biogenesis, cell wall membrane envelope biogenesis, coenzyme transport and metabolism, signal transduction mechanisms, amino acid transport, and inorganic ion transport and metabolism (Fig. S2). Some DEGs were, however, associated with virulence; the functions of these genes were previously evaluated in functional genomic analyses (5, 6) (Fig. 2B and Table S2).

A significant upregulation (log FC > 3; *P* value < 0.05; *FDR* < 0.05) of genes putatively encoding proteins associated with lipopolysaccharide (LPS) O-antigen biosynthesis, and therefore potentially linked to host cell adhesion (6), was observed in strain LMG 11119. These genes (*lacA*, *cotSA*, *pgaB*, *sunS*, *rfaG*, *mdoB*, and the putative O-antigen ligase-encoding gene) were upregulated jointly with *macA* and two copies of *macB*. The proteins encoded by *macA* and *macB* have been linked to the transmembrane transport of LPS and similar glycolipids and to the virulence and antibiotic resistance of Gram-negative bacteria (17). Moreover, other genes associated with LPS metabolism (*rmlA*, *rmlC*, *rmlD*, *gmhA1*, *rfaF*, and *gmhB*) were also differentially expressed, albeit less strongly (log FC = 1.5 to 3).

Genes currently considered to be associated with A. butzleri virulence and linked to chemotaxis (*cheD*, *cheY*, *cheR*, and *mcp4*) or two-component regulatory systems (*phoP*, *phoQ*, *mprA*, and *ttrS*) (5, 6) were upregulated in strain LMG 11119. In addition, the genes *exbB* and *exbD*, which are linked to TonB protein activity (involved in virulence and iron transport) (5) and are adjacent in the genome, were upregulated in LMG 11119. Finally, the genes *mviN* (*murJ*), *tlyA*, and *cadF* (*oprF*) were also upregulated in strain LMG 11119; the proteins encoded by these genes are considered *Arcobacter* virulence factors (5–7). Overall, the gene upregulation pattern observed in strain LMG 11119 was in accordance with previous observations regarding the virulence behavior of other Gram-negative bacteria. Studies on Escherichia coli (18) and Salmonella enterica (19) reported an increase in the expression of virulence genes when the bacteria came into contact with DMEM or Eagle’s minimal essential medium (MEM). Moreover, the transfer of bacteria from *Arcobacter* medium to DMEM, which has a different composition (in terms of sugars, amino acids, salts, and pH), together with the transition from a solid to a liquid medium, may explain the need to modulate gene expression in response to new environmental conditions (19–21). The rapid response to changing environmental conditions exhibited by strain LMG 11119 could also activate putative virulence genes, which may also be advantageous during infection and passage through the gut.

### Evaluating the expression of potential virulence genes in A. butzleri during host cell infection.

**FIG 3:**
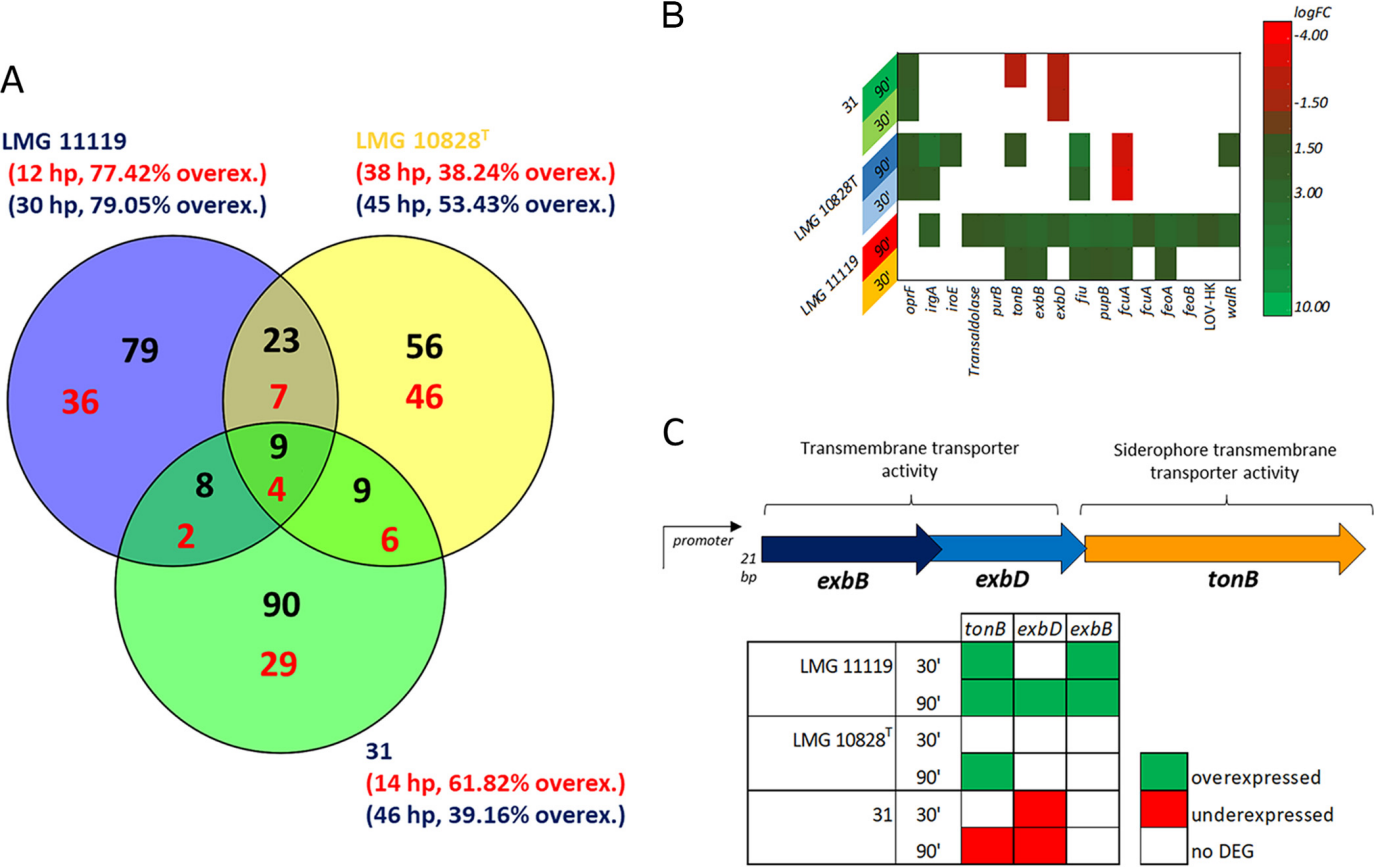
Venn diagram of DEGs in the A. butzleri strains (A); heat map of virulence-related DEGs in A. butzleri after contact with host cells (B); *tonB* operon organization (C). The Venn diagram shows the DEGs shared between different strains of A. butzleri and DEGs present in each single strain after 30 (red) or 90 (black) min of contact with mucus-producing human host cells. Hypothetical proteins were excluded, and the percentages of upregulated genes (calculated using the total number of DEGs, including hypothetical proteins) are indicated in parentheses below the strain name (red = 30 min; blue = 90 min). The heat map shows DEGs in A. butzleri strains after 30 or 90 min of contact with host cells, focusing on putative A. butzleri virulence genes. In the table in panel C, the upregulation (green) or downregulation (red) of the indicated gene is shown at the 30- and 90-min time points. A putative promoter was detected 21 bp upstream of the *exbB* gene.

In all three A. butzleri strains, DEGs were detected after 30 or 90 min of contact with the mucosal monolayer. Strain LMG 11119, characterized by a higher human cell colonization ability, had a higher ratio of upregulated DEGs (48/62 at 30 min and 128/161 at 90 min). The ratio of upregulated DEGs was lower in LMG 10828^T^ (39/102 at 30 min and 78/146 at 90 min) and 31 (34/55 at 30 min and 65/166 at 90 min), indicating that in these strains, a higher number of DEGs was downregulated. As most of these DEGs were specific to each strain, the number of DEGs shared between the three strains was relatively small (Fig. 3A and Tables S3 and S4). The majority of the DEGs (both up- and downregulated) were linked to energy production and conversion (Fig. S3). However, a few A. butzleri putative virulence genes were also among the DEGs (Fig. 3B).

We observed the upregulation of some of the 10 putative virulence genes, which are used in the detection of virulence traits in different *Arcobacteraceae* species (7, 22). The genes *oprF* (also called *cadF*; associated with host cell adhesion) and *irgA* (involved in iron metabolism) (7) were upregulated at the 30-min time point. Moreover, *iroE* (iron acquisition and infection maintenance) was upregulated in strain LMG 10828^T^ at the 90-min time point. The *oprF* gene was also upregulated in strain 31 (but not in LMG 11119) at both the 30- and 90-min time points.

We found that regulatory genes related to bacterial virulence were differentially expressed by the A. butzleri strains. The gene *walR*, which forms part of a two-component regulatory system linked to Staphylococcus virulence (23), and a gene encoding a histidine kinase (LOV-HK), which is associated with Brucella virulence (24), are adjacent in LMG 11119 genome; both genes were upregulated after 90 min of host cell contract.

A transaldolase-related gene was upregulated at the 90-min time point in strain LMG 11119. This enzyme is linked to the bacterial colonization of host mucosa (13). The upregulation of this gene in the strain characterized by the higher colonizing ability (i.e., LMG 11119) of mucus-secreting cells supports the notion that this is a virulence gene.

### Upregulation of TonB complex-associated genes is linked to higher A. butzleri colonizing ability.

The TonB complex is composed of the TonB, ExbB, and ExbD proteins; it is attached to the inner bacterial membrane and is involved in iron transport and assimilation. The TonB protein, a siderophore transport protein linked to the outer membrane, is essential for TonB complex function (25). The genes *exbB*, *exbD*, and *tonB* are adjacent in the A. butzleri genome and are preceded by a putative promoter upstream of *exbB* (Fig. 3C).

In strain LMG 11119, the upregulation of *tonB* and *exbB* was observed at the 30-min time point, while *exbD* was not differentially expressed. After 90 min of host cell contract, all three TonB complex-encoding genes were upregulated in strain LMG 11119. In contrast, strain LMG 10828^T^ showed upregulation only of the *tonB* gene (and only at 90 min), while in strain 31, both *tonB* (at 90 min) and *exbD* (at 30 and 90 min) were downregulated. Other genes related to the TonB complex were also differentially expressed. For instance, *fiu* (which encodes the catecholate siderophore receptor, Fiu) was upregulated at the 30- and 90-min time points in strains LMG 11119 and LMG 10828^T^. The gene *fiu* has been linked to the catecholate siderophore-mediated active transport of complexed iron across the outer cell membrane; this gene is therefore considered linked to TonB complex function (26). Other genes encoding siderophore-related proteins linked to iron assimilation were also upregulated in strain LMG 11119. These genes included *pupB* (encoding the siderophore ferric pseudobactin receptor, BN8), *fcuA* (encoding a ferrichrome receptor), *feoA* (encoding a Fe^2+^ uptake protein), and *feoB* (encoding a putative Fe^2+^ uptake protein) (27). We found that *pupB*, *fcuA*, and *feoA* were upregulated at 30 and 90 min, while *feoB* was upregulated at 90 min only. In strain LMG 10828^T^, *fcuA* was downregulated at both time points. The gene *irgA* (encoding an iron-regulated outer membrane virulence protein), which is linked to iron assimilation (26, 28), was upregulated at 30 and 90 min in strain LMG 10828^T^ and at 90 min in strain LMG 11119.

These findings highlight the important roles of genes related to TonB complex function and iron assimilation in A. butzleri pathogenesis. The expression of these TonB complex-associated genes was also related to physiological differences between A. butzleri strains. The gene expression patterns that we observed were in accordance with previous studies of TonB in other Gram-negative bacteria, such as E. coli and Pseudomonas plecoglossicida, where TonB complex mutants attenuated virulence (29, 30). Furthermore, iron transport and uptake are considered important in several pathogenic bacteria (31, 32), including Yersinia pestis, which produces siderophores in an iron uptake system (33, 34). In addition, the deletion of the ferric uptake regulator, Fur, reduced the virulence of the avian pathogen Riemerella anatipestifer (35). Finally, a mouse study showed that Salmonella responds to iron-depleting conditions in the host by upregulating genes involved in iron acquisition (36). It is, however, important to remember that a small amount of iron is present in DMEM (at a concentration of 0.25 μM) in the form of ferric nitrate (37). Exposure of A. butzleri strains to ferric nitrate in DMEM could have contributed to the activation of iron transport-related genes. However, results obtained from the study of other pathogenic bacteria (29, 30, 38) suggest that iron transport and assimilation have important roles in A. butzleri virulence.

### Differential expression of genes associated with organic acid metabolism.

While A. butzleri strains can metabolize lactate, they are generally not able to metabolize acetate. Strain ED-1 is the exception, as it can grow in the presence of lactate and acetate and moderately in the presence of succinate. Therefore, A. butzleri strains vary in terms of their organic acid metabolism (39, 40). The metabolic pathways of human cells are also influenced by acetate and lactate concentrations (41). In human cells, an increase in lactate concentration has been linked to the induction of the stress response (42); however, human cells can also metabolize lactate (43). Acetate is the main substrate that supports acetyl coenzyme A (acetyl-CoA) metabolism in human cells (41, 44), while its consumption under stress conditions has also been observed (45).

**FIG 4:**
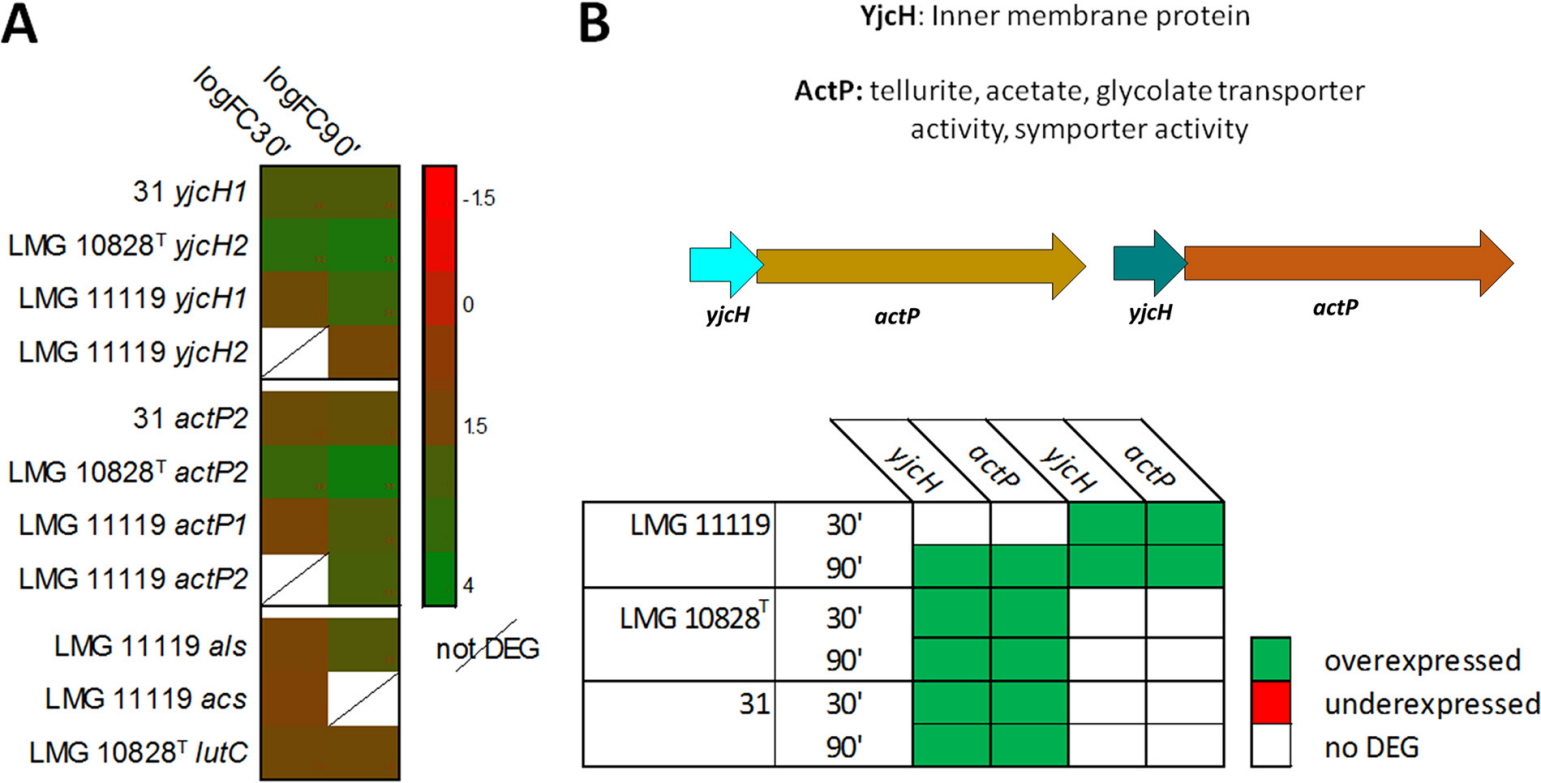
A. butzleri DEGs related to acetic acid and lactic acid metabolism. The heat map in panel A shows A. butzleri DEGs associated with the metabolism of lactate and acetate during host cell infection (*P* value < 0.05; FDR corrected).The association between *actP* and *yjcH* expression is shown in panel B; both genes were upregulated (green) at the 30- and 90-min time points.

The genome of A. butzleri contains genes linked to the metabolism of acetate and lactate (39). Moreover, we found that some of these genes were differentially expressed (e.g., *yjcH* and *actP*) after exposure to the host cell *in vitro* model (Fig. 4A and B). Different gene forms can coexist in the same genome. These genes present differences at the sequence level, maintaining characterizing protein domain sequence and a protein similarity greater than 30% (coverage > 95%). The *yjcH* gene (encoding the inner membrane protein YjcH), which is involved in acetate dissimilation and transport, and the *actP* gene (encoding the cation/acetate symporter ActP), which is linked to acetate transport in E. coli (46), were generally upregulated at 30 and 90 min in the three A. butzleri strains. In strain LMG 11119, the gene *actP* was downregulated at 30 min (log FC = −1.49); however, the expression of both *actP* copies was upregulated at 90 min. The gene *acs* (encoding an acetyl-CoA synthetase, Acs) was differentially expressed only at 30 min in strain LMG 11119, where it was only weakly upregulated (log FC = 1.37). The Acs protein catalyzes the reaction which converts acetate, coenzyme A, and ATP into AMP, pyrophosphate, and acetyl-CoA (47). In E. coli, *acs* forms an operon with *actP* and *yjcH* (46), while in A. butzleri, it is present in a genomic region distant from *actP* and *yjcH*. In this study, we found that the expression of one copy of each of the *yjcH* and *actP* genes was upregulated at 30 and 90 min in strains 31 and LMG 10828^T^, while the expression of both gene copies was upregulated at 90 min in strain LMG 11119.

**FIG 5:**
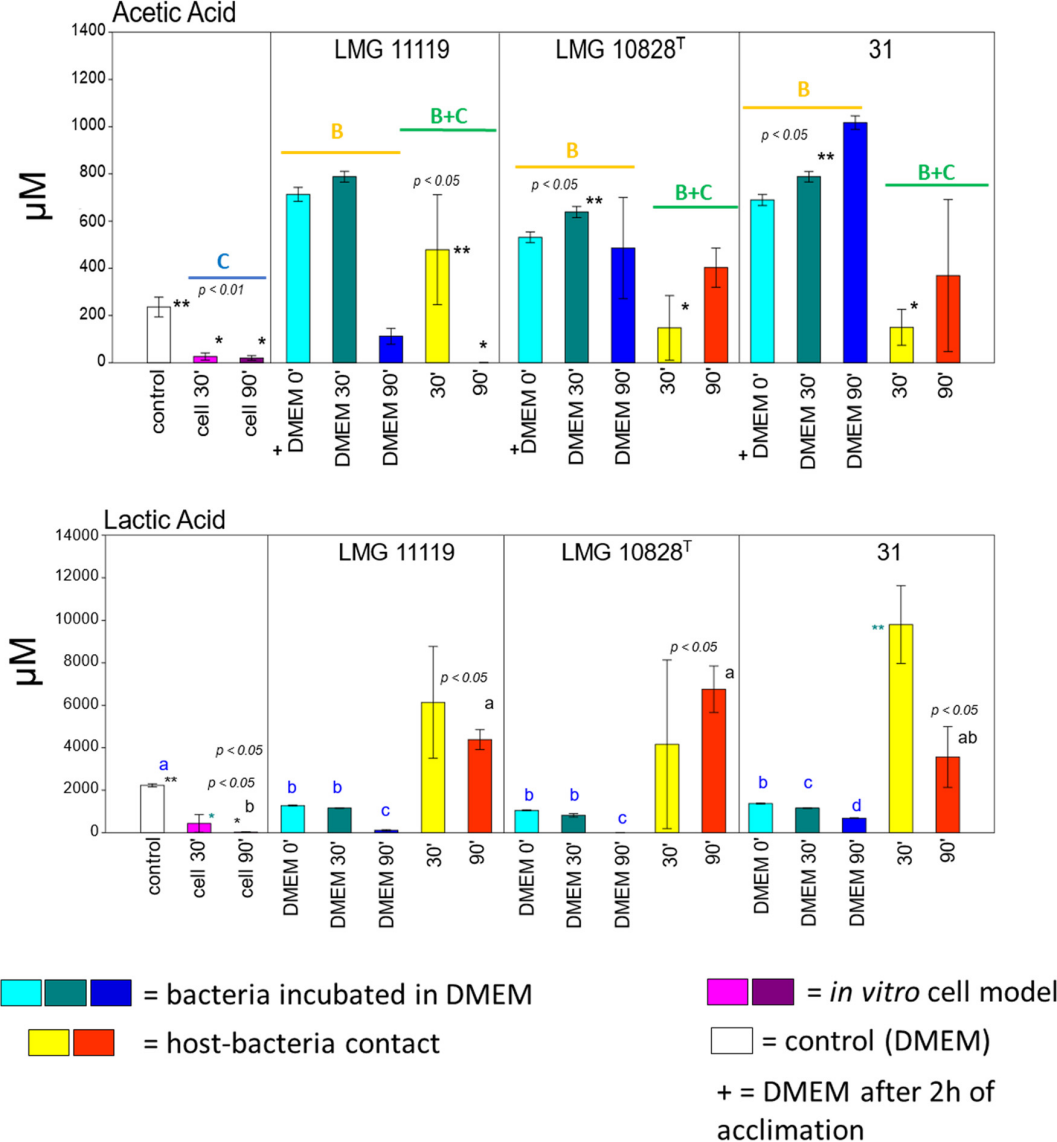
Analysis of acetic and lactic acid concentrations in the culture medium. The bar charts show the concentrations of acetic and lactic acid in the DMEM; the A. butzleri strain names and sampling times of 0 (after 2 h of adaptation), 30, and 90 min are indicated. The different conditions are indicated by different colors (see color key), while the colored lines above the bars show comparisons between the following conditions: host cell culture in DMEM (blue; C), bacterial culture in DMEM (yellow; B), and bacterial culture with host cells in DMEM (green; B and C). The error bars represent the SEMs, while the *P* values indicate the statistical significance of differences between conditions (the different letters and colors are related to different comparisons).

We therefore decided to measure the concentration of acetate in the culture medium under different test conditions (Fig. 5). The chemical analyses performed on DMEM obtained from different culture conditions confirm changes in the expression of organic acid-related genes (Fig. 4A and B). Acetate was not detectable in DMEM after 90 min of host cell-LMG 11119 contact. At the 30-min time point, some acetate was present in the medium; however, the concentration of acetate was lower than that of the DMEM adaptation-phase samples (*P* value < 0.02). For the other two strains, the differences in acetate concentrations between the 30- and 90-min time points were not significant (*P* value > 0.05). The decrease in acetate concentration over the course of infection with LMG 11119, but not the other strains, may explain why this strain also had a larger number of DEGs associated with organic acid metabolism. This result is reminiscent of the observation in E. coli whereby the operon *acs-yjcH-actP* was involved in the acetate assimilation system and enabled E. coli to replicate inside macrophages (46). In an avian study of lung infection, the deletion of this operon reduced E. coli virulence and colonizing ability (46). Moreover, the presence of acetate promoted E. coli adherence to Caco-2 cells and increased its motility on semisolid LB agar plates (48). A correlation between acetate metabolism and adherent-invasive E. coli (AIEC) was also observed by Elhenawy and colleagues (49). The authors hypothesized that acetate could be used as an alternate energy source by AIEC, giving it a metabolic advantage (49). Thus, findings from studies of E. coli and the data from this study collectively imply that acetate consumption is important for the survival and virulence of A. butzleri.

We therefore decided to compare the compositions of DMEM after 30 min of bacterium-host cell coculture and DMEM containing host cells alone. We found that acetate concentration increased 18.54-fold after 30 min of infection with strain LMG 11119 (*P* value < 0.05). The increase in acetate concentration after 30 min of infection suggests that the host stress response was activated during the bacterial colonization phase (50). The acetate concentration after 30 min of infection with strain 31 or LMG 10828^T^ was significantly lower than in the DMEM adaptation condition (an 81% or 76.91% decrease, respectively; *P* value < 0.03). We then compared acetate concentrations between A. butzleri plus DMEM and DMEM alone and found that the acetic acid concentration increased after bacterial adaptation, suggesting that acetic acid was being produced by the three A. butzleri strains (Fig. 5). This shows that A. butzleri can produce as well as consume acetic acid (40). It is important to note that in this study, acetate could have also been produced by host cells or could have already been present in DMEM supplemented with fetal bovine serum (FBS) (51). Moreover, as previously stated, human cells are also capable of consuming acetate (41) (Fig. 5).

An increase in lactic acid concentration was observed after 90 min of infection with the LMG 11119 and LMG 10828^T^ strains, compared with the case with DMEM plus A. butzleri (*P* value < 0.03) (Fig. 5). The lactic acid concentration after 90 min of infection was higher for strains LMG 11119 and LMG 10828^T^ than when the human cell line was cultured with DMEM alone (average increase = 310-fold; *P* value < 0.01). Similarly, infection with strain 31 increased lactic acid levels at the 30-min time point (average increase = 22.79-fold; *P* value < 0.01) (Fig. 5). The fact that the increase in lactic acid concentration was not accompanied by relevant DEGs suggests that it was produced by host cells and not A. butzleri. This has already been observed in the Caco-2 cell line in response to E. coli infection (52). The lactic acid concentration also decreased at the 90-min time point when bacteria were culture in DMEM without host cells, confirming the ability of A. butzleri to consume lactate (40, 53). In this instance, lactate likely originated from the FBS present in DMEM (54) (*P* value < 0.01) (Fig. 5).

We found that the glucose concentration did not change significantly in DMEM plus bacteria versus uninoculated DMEM, except for a decrease at the 90-min time point for strain LMG 11119 (13.53% reduction in the starting glucose concentration; *P* value < 0.01). Moreover, when strain 31 was incubated in DMEM, the glucose concentration decreased only slightly at the 90-min point (4.36% reduction in the starting glucose concentration; *P* value < 0.01) (Fig. S4). These findings are in accordance with the low number of DEGs (average = 2.46%) associated with carbohydrate transport and metabolism detected in the three strains placed under different conditions (Fig. S2 and S3 and Table S5). The lack of glucose fermentation has already been reported for A. butzleri (40, 53), while differences in glucose consumption among strains have been observed in Campylobacter species (2, 55).

The consumption of pyruvic acid by A. butzleri was more pronounced than that of glucose. A decrease in pyruvic acid concentration was observed when the bacteria were transferred to DMEM and incubated for 90 min. Pyruvate concentration decreases of 99.16% and 21.43% were observed upon transferring strains LMG 11119 and 31, respectively (*P* value < 0.001), for both the 30- and 90-min time points. A 50.01% decrease in pyruvic acid concentration was observed when strain LMG 10828^T^ was incubated with DMEM for 90 min (*P* value < 0.01); no statistically significant differences in pyruvate concentration were observed at the 30-min time point (Fig. S4). These results are in accordance with the ability of A. butzleri to consume pyruvic acid, which has already been reported (53).

### Conclusions.

Recent studies (5, 6) on A. butzleri have provided valuable information regarding the genomic characteristics of this bacterium. In the present study, we performed a transcriptome analysis of three physiologically different A. butzleri strains using the mucus-producing human intestinal *in vitro* model. The colonizing ability of these strains (in the absence of higher invasiveness) was confirmed, together with the involvement of some putative virulence genes. We found that strain LMG 11119 had higher colonizing ability and responded rapidly to new environmental stimuli. The rapid expression of genes in response to environmental cues by A. butzleri could play a role in its adaptation to the host as well as its virulence mechanism.

The upregulation of genes linked to iron transport in the A. butzleri strain characterized by higher colonizing ability (i.e., LMG 11119) is also relevant. The upregulation of these genes implies that iron metabolism is important during A. butzleri infection. Other DEGs that appeared to be important were involved in organic acid metabolism; however, this has already been observed in other Gram-negative bacteria (46, 49). The expression pattern of these genes, coupled with the changes in organic acid concentrations that were observed in this study, suggests that the consumption of host-derived organic acids was greatest by the strain that showed highest colonizing ability. Moreover, the expression of genes related to organic acid metabolism correlated well with the consumption of organic acids by A. butzleri during the colonization of host cell lines. Moreover, we hypothesize that A. butzleri infection induced a stress response in the host cells, which led to the increased release of lactate by human intestinal cells. Future studies will be needed to consolidate the role of organic acid metabolism in the virulence of A. butzleri and the subsequent host-mediated response. The data presented here highlight the importance of functional genome annotation. Additionally, we provided further evidence regarding the involvement of genes that were already considered virulence related. Even though we identified a considerable number of genetic sequences with an unknown function, we were able to attribute a role of some of these genes in the A. butzleri virulence process. Although this work was performed using a simplified *in vitro* model, the data can be used to inform subsequent targeted *in vitro* and *in vivo* studies, as well as propose the detection of these newly identified genetic sequences in A. butzleri-contaminated foods and clinical samples.

## MATERIALS AND METHODS

### A. butzleri culture and bacterial suspension preparation.

The A. butzleri strains used in this study were isolated from human stool samples, which were collected from different geographical areas: LMG 10828^T^ (United States), LMG 11119 (Italy), and 31 (Belgium). All three strains were obtained from the Belgian Coordinated Collection of Microorganisms (BCCM; Laboratory for Microbiology, Ghent University, Belgium) and cultured under microaerophilic conditions at 30°C on *Arcobacter* agar (CM0965; Oxoid, Basingstoke, UK), supplemented with C.A.T. (SR0174; Oxoid).

The bacterial pellets were collected for each experiment using the following method. An A. butzleri colony was inoculated into 5 mL of *Arcobacter* broth and incubated at 30°C for 48 h. Next, 500 μL of culture was inoculated onto *Arcobacter* agar plates supplemented with C.A.T. After 48 h of incubation under microaerobic conditions, the strains were collected with 1 mL of Ringer’s solution (1.15525; Millipore, Burlington, MA, USA). After two washing steps with Ringer’s solution (by centrifugation at a relative centrifugal force [RCF] of 16,000 for 10 min), the approximate bacterial load of each working suspension was determined by optical density (OD) evaluation at 630 nm on an ELx880 microtiter plate reader (Savatec, Turin, Italy) using an internal standard curve. The bacterial suspension used for the *in vitro* colonization tests and transcriptomic analyses was prepared in Dulbecco’s modified Eagle’s medium (DMEM; 6429; Sigma-Aldrich, St. Louis, MO, USA) supplemented with 10% fetal bovine serum (FBS; F7524; Sigma-Aldrich).

### Generation of the mucus-producing human intestinal *in vitro* model and cell culture.

Two human colon carcinoma cell lines, Caco-2 (86010202; European Collection of Authenticated Cell Cultures [ECACC], Public Health England) and HT29-MTX-E12 (12040401; ECACC), were cultured in DMEM (6429; Sigma-Aldrich, St. Louis, MO, USA) supplemented with 10% FBS (F7524; Sigma-Aldrich) and EmbryoMax penicillin-streptomycin solution (TMS-AB2-C; Sigma-Aldrich). The cell lines were grown in culture flasks (Corning, New York, NY, USA) at 37°C in a humidified atmosphere (5% CO_2_) and subpassaged every 3 to 4 days (Galaxy 170 S; Eppendorf, Hamburg, Germany). The mucus-producing epithelial monolayer was prepared by coculture of Caco-2 and HT29-MTX-E12 cell lines, combined in a 9:1 ratio (11). Briefly, the cells were seeded at a density of 35,000 cells cm^−2^ on 21.2-cm^2^ cell culture plates to obtain sufficient material for RNA analysis and on 1.93-cm^2^ plates for studies of A. butzleri colonization-invasion. The cells were grown in complete culture medium under the same conditions as described above for 14 to 15 days. On reaching functional polarization, the cells were considered fully differentiated, and the model could be used in experiments (56). Three days before the *in vitro* experiments, the cell monolayer was washed twice with phosphate-buffered saline (PBS) to remove traces of antibiotics. After the washing step, the culture medium was replaced with the same medium lacking antibiotics to enable bacterial growth.

### *In vitro* colonization-invasion assay.

The *in vitro* colonization-invasion assay was performed as previously described, with some modifications (6). Briefly, the three A. butzleri strains were collected from *Arcobacter* agar plates, resuspended in DMEM, and incubated for 2 h (37°C, 5% CO_2_). After this acclimation period, the DMEM-cultured A. butzleri at time zero (T0) was used as a control for the transcriptome analysis. The A. butzleri strains in DMEM at T0 were then inoculated onto the *in vitro* mucus producer human cells by replacing three-fourths of the model medium with the bacterial suspension; an average load of log 6.54 CFU cm^−2^ (standard error of the mean [SEM] = 0.29) was applied (Table S1). Colonization-invasion was evaluated in parallel after 30 or 90 min of bacterial infection. For each infection period, experiments were performed in three biological replicates using samples from two different model wells. After 30 or 90 min of bacterium-host cell contact at 37°C (5% CO_2_), any free bacteria were removed by two washes with PBS. The colonization ability (i.e., the ability of bacteria to adhere to and enter host cells [T1]) of each A. butzleri strain was evaluated by treating each cell model well with 0.5 mL of 0.25% Triton X-100 (vol/vol, in PBS). After 30 min of incubation at 37°C, the resulting suspension was analyzed to evaluate the colonizing bacterial load (the CFU method) by serially diluting the samples in Ringer’s solution and plating them onto C.A.T.-supplemented *Arcobacter* agar medium. The bacterial load was evaluated after 48 h of incubation at 30°C under microaerobic conditions. The A. butzleri invasion capability (i.e., a measure of the number of bacterial cells that gained entry into a host cell [T2]) was evaluated in parallel by adding 0.5 mL of DMEM (without FBS) containing 300 μg mL^−1^ of gentamicin sulfate (G1914; Sigma-Aldrich) to kill all the bacteria outside the host cells. After 2 h of gentamicin treatment at 37°C, two PBS washing steps were performed to eliminate traces of the antibiotic. The load of internalized A. butzleri was evaluated using the CFU methods described above. The raw count data were expressed as log CFU per square centimeter. The colonization load was calculated as T1 log CFU cm^−2^ – T0 log CFU cm^−2^, while the load of internalized bacteria was calculated as T2 log CFU cm^−2^ – T0 log CFU cm^−2^.

During the *in vitro* tests, three biological sample replicates were taken from each well and used for transcriptome analysis. These samples were taken under the same conditions as the colonization-invasion tests previously exposed. Briefly, after 30 or 90 min of A. butzleri infection, on human cell models (21.2-cm^2^ cell dishes), the *in vitro* intestinal cell monolayer was washed twice with PBS. The cell layer containing adhered and internalized bacteria was then collected into 1 mL of PBS and centrifuged at an RCF of 16,000 for 5 min at 4°C. The PBS was then removed and 0.5 mL of RNAlater (AM7021; Invitrogen; Waltham, MA, USA) was added to the pellet, which was subsequently stored at −20°C for subsequent analyses. RNAlater was also added to 1 mL of A. butzleri DMEM suspension after adaptation (produced as described above) and to 1 mL of A. butzleri suspension collected from *Arcobacter* agar (by centrifugation at an RCF of 16,000 for 5 min at 4°C) before adaptation; these samples were used as controls.

### Genome sequencing and bioinformatics analysis.

Genomic DNA was extracted from A. butzleri strains using the bead-beating, phenol-chloroform-isoamyl alcohol DNA extraction method, followed by RNase A (5 μg μL^−1^, MRNA092; Epicenter, Madison, WI, USA) treatment by incubation at 37°C for 30 min to digest any contaminating RNA.

The DNA concentration was quantified using a NanoDrop instrument (ND1000; Thermo Fisher Scientific) and separated (100 V, 30 min) on a 0.8% agarose gel (wt/vol; 0710; VWR) in TAE buffer (containing Tris, acetic acid, and EDTA; K915; VWR) with gelRed (41003; Biotium, Fremont, CA, USA) as the DNA intercalating agent. This electrophoretic run was performed as a DNA quality check. Illumina NovaSeq 6000 whole-genome sequencing (paired end, 150 bp; coverage, 100×) was performed by Novogene (Cambridge, UK) after Qubit 2.0 quantification. One microgram of genomic DNA (gDNA) was used for library preparation (New England BioLabs [NEB], Ipswich, MA, USA). The gDNA was randomly fragmented by shearing into ~350-bp fragments, followed by polishing. The fragments were then A-tailed, ligated to the NEBNext adapter, and PCR amplified (using P5 and P7 indexed oligonucleotides). PCR product purification was performed on an AMPure XP system. The libraries were analyzed on an Agilent 2100 Bioanalyzer (size distribution evaluation) and quantified using real-time PCR.

### RNA-seq.

RNA sequencing (RNA-seq) was performed by GENEWIZ, LLC (South Plainfield, NJ, USA) on RNA extracted from A. butzleri-infected intestinal cells as described in the relevant section. Total RNA was extracted with the Qiagen RNeasy Plus universal minikit, following the manufacturer’s instructions (73404; Qiagen, Hilden, Germany). The samples were quantified on a Qubit 2.0 fluorometer (Life Technologies, Carlsbad, CA, USA), and the RNA integrity was evaluated on a 4200 TapeStation (Agilent Technologies, Palo Alto, CA, USA). rRNA depletion was performed using the FastSelect rRNA H/M/R kit (334385; Qiagen). The sequencing libraries were then prepared using the NEBNext Ultra RNA library prep kit for Illumina, following the manufacturer’s instructions (E7770; NEB, Ipswich, MA, USA). The enriched RNA was fragmented for 15 min at 94°C. Next, first- and second-strand copy DNA was synthesized, end repaired, and adenylated to form 3′ ends. After adenylation, the universal adapter was ligated to the copy DNA fragments, followed by indexing and PCR library enrichment. The resulting libraries were validated using Agilent TapeStation 4200 (Agilent Technologies) and quantified using a Qubit 2.0 fluorometer and quantitative PCR (Applied Biosystems, Carlsbad, CA, USA). The sequencing libraries were multiplexed and clustered onto the flow cell, prior to being sequenced on an Illumina NovaSeq 6000 system. Samples were sequenced to a depth of ~20 million read pairs per sample, while ~40 million read pairs per sample were generated for human epithelial cells plus A. butzleri (2 × paired end, 150 bp). Image analysis and base calling were performed using the NovaSeq Control software v1.6; raw sequence data (.bcl format) obtained from the NovaSeq instrument were converted into fastq files and demultiplexed using the Illumina bcl2fastq program v2.20 (one mismatch was tolerated for index sequence identification).

### Genome and transcriptome bioinformatics and statistical analysis.

The whole-genome sequencing reads were quality filtered with Solexa QA software (57). PRINSEQ++ v1.2 (58) was then used to remove sequences shorter than 60 bp and dereplicated sequences. The reads were *de novo* assembled at our laboratory using SPAdes software v3.11.0 (59). The contigs were quality screened using Quality Assessment Tool for Genome Assemblies (QUAST) v5.0.0 (60) and annotated using the Prokka tool v1.11 (61) (Table S6). Promoter detection was performed using the BPROM (62) and BDGP (63) tools. Graphical visualization of the genomes was performed using Anvi’o software v7.1 (mcl 10) (64). Gene enrichment analysis was performed using the emapper v2.1.6 tool (65), while SWISS-MODEL (66) was employed to detect specific genetic sequences within the differentially expressed genes (DEGs) of interest.

Analysis of the A. butzleri transcriptome was performed as follows. Raw read data were quality checked with QUAST v5.0.0 (60) and the adapters were removed with cutadapt v3.1 (67). Raw reads were next quality filtered using SolexaQA++ v3.1.7.1 (57) and PRINSEQ v0.20.4 (58) (Phred score < 20; <51 bp). Reads associated with human contaminants were discarded using Bowtie 2 v2.3.5 in end-to-end, sensitive mode (68). Clean reads were then aligned against an annotated A. butzleri strain using Bowtie2, in end-to-end, sensitive mode. Information on the number of reads mapped to each gene was extracted using samtools v1.2. The raw transcript count table was normalized using the reads per kilobase of transcript per million reads mapped method (RPKM), taking into consideration the experimental conditions and the characteristics of the obtained transcriptome libraries (69). The normalized transcript count data were interrogated using EdgeR Bioconductor R package v3.14 to identify possible DEGs (70, 71). Genes characterized by log FC (*P* value < 0.05;, FDR < 0.05) of ≧1.5 or ≦−1.5 were considered DEGs; exceptions to these values are indicated.

### HPLC.

The DMEM samples obtained from A. butzleri and cell model incubation and coincubation were centrifuged at an RCF of 16,000 for 10 min at 4°C. The supernatant was then sterile filtered (0.2 μm) and chemically analyzed by high-performance liquid chromatography (HPLC). The HPLC system (Thermo Electron Corporation, Waltham, MA, USA) was equipped with an SCM 1000 degasser, a P2000 binary gradient pump, a multiple autoinjector (AS3000), a photodiode array (PDA) detector (UV6000LP; Thermo Electron Corporation), and a refractive index detector (RI-150). The mobile phase was 0.013 N H_2_SO_4_ and the sample injection volume was 20 μL. The isocratic elution method was applied with a flow rate of 0.6 mL min^−1^ for 30 min. The samples were loaded onto a reverse-phase Aminex HPX-87H column (300 by 7.8 mm) equipped with a Microguard cartridge (Bio-Rad Laboratories, Hercules, CA, USA), working at 65°C. Data analysis was performed using the ChromQuest chromatography data system (ThermoQuest software 5.0; Inc., San Jose, CA, USA), while protein identification was achieved by comparison with the retention times of standards supplied by Sigma-Aldrich (Milan, Italy).

### Statistical analysis.

Statistical analysis was performed using RStudio software v2021.09.0 (R 3.6.1 [https://www.r-project.org/]). Shapiro-Wilk’s W and modified Levene’s tests (i.e., the Brown-Forsythe test) were respectively used to check data normality and homogeneity. The Kruskal-Wallis (K-W) test and analysis of variance (ANOVA) were used to evaluate overall differences and variations between multiple groups, while the Wilcoxon rank sum test (WRS) and the two-sample *t* test were used to evaluate differences between two groups. These tests were used for nonparametric (K-W and WRS) and parametric (ANOVA and *t* test) data. The Dunn’s and Tukey’s tests were used in *post hoc* analyses of nonparametric and parametric data, respectively. Bar charts (displaying means ± SEMs) were generated using Past3 software v3.17 (72).

### Data availability.

Raw whole-genome sequence read data were deposited into the Sequence Read Archive of the National Center for Biotechnology Information using accession numbers SRX9057116 (LMG 10828^T^), SRX9057105 (LMG 11119), and SRX9057128 (31). The RNA-seq raw data are available under BioProject number PRJNA703833 (3 = LMG 10828^T^; 2 = LMG 11119 and 31). The assembled genomic sequences, functional annotation files, and log FC table are available on Zenodo (https://zenodo.org/record/5882246#.Y2pVK-zP1hA).
