# Supplementary material for: Transcriptome Analysis of Arcobacter butzleri Infection in a Mucus-Producing Human Intestinal In Vitro Model
Source: Microbiol Spectr. 2023 Jan 9;11(1):e02071-22. doi: 10.1128/spectrum.02071-22 (PMC9927503; doi:10.1128/spectrum.02071-22)
Supplement: Supplemental file 1 — Supplemental material. Download spectrum.02071-22-s0001.pdf, PDF file, 1.1 MB [file spectrum.02071-22-s0001.pdf]

Supplementary Table 1. *A. butzleri in vitro* test log CFU cm^{-2} values. The data in this table represent bacteria load of bacterial inoculum (T0), bacteria detected after PBS washing (T1) and bacteria detected after gentamicin application (T2). T1 and T2 loads have been detected at 30 minutes (30') and 90 minutes (90'). In table are shown the corresponding standard errors while "ND" indicates a not detectable load.

strain	LMG 11119	LMG 10828^T	31
**T0**			
average (log)	6.58	6.54	6.51
standard error	0.15	0.09	0.63
**T1 30'**			
average (log)	5.42	3.82	2.25
standard error	0.04	0.22	1.14
**T1 90'**			
average (log)	6.21	3.76	3.90
standard error	0.53	0.20	0.67
**T2 30'**			
average (log)	3.64	ND	1.91
standard error	0.03	ND	0.99
**T2 90'**			
average (log)	3.68	2.68	1.71
standard error	0.26	0.13	0.86

Supplementary Table 2. *A. butzleri* DMEM DEGs from the comparison with Arcobacter agar. The table shows logFC (< -1.5, > 1.5) logCPM, *p value* (< 0.05) and FDR (< 0.05) values of differentially expressed genes linked to currently considered putative virulence genes. The column "gene" shows the protein name of the DEGs and the relative locus tag. The name of the genes indicated in the main text is indicated in brackets after the name of the protein.

gene	logFC	logCPM	P value	FDR
Strain LMG 11119				
01097 Virulence sensor protein BvgS precursor	2.25	3.29	1.31E-09	1.83E-09
00898 Capsule polysaccharide biosynthesis protein	2.18	5.09	2.95E-18	5.45E-18
00852 Chemotaxis response regulator protein-glutamate methylesterase	2.75	5.95	1.07E-32	3.54E-32
00851 Chemoreceptor glutamine deamidase CheD (*cheD*)	3.41	6.44	8.45E-51	7.37E-50
01001 Methyl-accepting chemotaxis protein IV	1.56	4.71	1.45E-10	2.09E-10
00850 Chemotaxis protein methyltransferase (*cheR*)	3.71	6.32	2.77E-49	2.14E-48
00201 Chemotaxis protein CheW (*cheW*)	2.32	8.70	2.80E-29	7.80E-29
00848 Chemotaxis protein CheY (*cheY*)	1.66	8.83	5.95E-16	1.01E-15
01806 Chemotaxis protein CheY (*cheY*)	3.79	7.90	8.55E-47	5.77E-46
00338 hypothetical protein	2.40	5.59	1.40E-24	3.26E-24

00605 hypothetical protein	2.03	6.65	1.44E-22	3.11E-22
00883 Colicin V production protein	4.38	7.06	3.87E-70	1.33E-68
02099 Spore coat protein SA (*cotSA*)	4.75	4.95	2.16E-45	1.36E-44
00429 Type II secretion system protein F	4.04	5.29	8.52E-39	3.78E-38
00892 Putative acetyltransferase EpsM	3.89	4.83	2.40E-35	8.91E-35
00380 Biopolymer transport protein ExbB (*exbB*)	3.75	7.51	4.15E-52	3.81E-51
00920 Biopolymer transport protein ExbB (*exbB*)	1.78	4.45	1.97E-10	2.82E-10
00155 Biopolymer transport protein ExbD (*exbD*)	4.74	6.05	9.52E-67	2.36E-65
00379 Biopolymer transport protein ExbD (*exbD*)	4.01	5.87	2.23E-41	1.14E-40
00919 Biopolymer transport protein ExbD (*exbD*)	1.80	5.99	1.75E-15	2.91E-15
02238 Sensor protein FixL	2.99	4.42	1.66E-19	3.18E-19
01554 Flagellum-specific ATP synthase	2.48	7.19	4.44E-25	1.06E-24
01686 Flagellar hook-associated protein 1	1.75	9.19	7.08E-16	1.19E-15
01531 Flagellar M-ring protein	3.81	5.73	1.83E-43	1.05E-42
01649 Flagellin N-methylase	3.62	5.51	6.18E-42	3.29E-41
01807 flagellar motor switch protein	3.84	6.97	2.31E-50	1.99E-49
01533 flagellar assembly protein H	2.37	5.15	1.08E-20	2.16E-20
01685 Flagellar L-ring protein precursor	3.12	6.66	3.79E-43	2.15E-42
01530 Flagellar basal body rod protein FlgB	2.98	6.43	1.64E-32	5.34E-32
01549 Flagellar basal-body rod protein FlgC	2.46	8.69	3.06E-30	8.95E-30
01529 Flagellar basal-body rod protein FlgG	2.13	7.36	1.48E-22	3.19E-22
01556 Flagellar biosynthesis protein FlhA	4.16	5.30	4.77E-42	2.57E-41
01552 Flagellar biosynthetic protein FlhB	3.19	6.01	2.13E-46	1.41E-45
01834 Flagellar biosynthetic protein FlhB	2.63	4.29	6.50E-17	1.14E-16
01545 Flagellar biosynthesis protein FlhF	3.89	5.75	6.01E-47	4.11E-46
01532 Flagellar motor switch protein FliG	3.17	5.65	2.40E-35	8.91E-35
01550 Flagellar hook-length control protein FliK	3.60	6.42	4.31E-45	2.64E-44
01833 Flagellar hook-length control protein FliK	1.87	6.14	3.38E-17	6.02E-17
01683 flagellar basal body-associated protein FliL	2.38	7.89	2.60E-21	5.34E-21
01677 Flagellar motor switch protein FliM	3.01	6.97	1.07E-40	5.34E-40
01541 Flagellar motor switch protein FliN	1.52	7.82	1.12E-12	1.70E-12
01066 Flagellar biosynthetic protein FliP precursor	3.54	5.48	4.89E-42	2.63E-41
01680 Flagellar biosynthetic protein FliQ	4.78	6.82	1.18E-82	1.92E-80
01551 flagellar biosynthesis protein FliR	3.71	4.96	2.34E-31	7.19E-31
01688 Flagellar protein FliS	3.47	8.32	5.67E-32	1.80E-31
00015 Flagellar assembly factor FliW 2	2.26	8.15	2.19E-16	3.76E-16
01103 HlyD family secretion protein	2.47	1.27	1.32E-02	1.48E-02
00729 Internalin-J precursor	1.85	6.25	1.27E-20	2.55E-20
00943 Colicin I receptor precursor (*cirA; irgA*)	1.98	1.70	2.97E-03	3.41E-03
02180 Chondroitin synthase	2.59	4.72	5.80E-19	1.08E-18
00285 Methyl-accepting chemotaxis protein 4	3.95	5.04	3.88E-20	7.59E-20
00174 Motility protein B	3.24	6.32	1.34E-41	7.00E-41
00229 Response regulator MprA (*mprA*)	3.48	4.92	1.17E-31	3.65E-31
00773 Response regulator MprA (*mprA*)	2.85	8.14	5.54E-16	9.37E-16
00783 Response regulator MprA (*mprA*)	2.97	3.90	9.01E-17	1.57E-16
01108 Response regulator MprA (*mprA*)	2.86	1.33	1.03E-03	1.19E-03
01367 Response regulator MprA (*mprA*)	1.84	8.87	1.43E-16	2.47E-16

01799 Response regulator MprA (*mprA*)	3.60	2.96	2.23E-12	3.36E-12
02088 Transcriptional repressor MprA (*mprA*)	2.25	5.06	6.01E-09	8.17E-09
01624 Lipid A export ATP-binding/permease protein MsbA	3.43	5.54	1.11E-36	4.47E-36
01623 putative peptidoglycan biosynthesis protein MurJ (*mviN*)	3.35	2.96	1.34E-12	2.03E-12
02097 O-Antigen ligase	3.34	2.96	4.65E-13	7.11E-13
00648 Outer membrane porin F precursor (*oprF; cadF*)	5.57	6.37	6.07E-88	1.54E-85
00828 putative sensor histidine kinase PdtaS	2.35	2.88	2.26E-07	2.92E-07
01219 putative sensor histidine kinase PdtaS	2.93	1.20	3.56E-03	4.08E-03
02101 Poly-beta-1,6-N-acetyl-D-glucosamine N-deacetylase precursor (*pgaB*)	4.21	4.57	3.05E-30	8.92E-30
02220 Virulence transcriptional regulatory protein PhoP (*phoP*)	1.93	6.25	3.92E-06	4.89E-06
02256 Transcriptional regulatory protein PhoP (*phoP*)	4.70	4.16	5.84E-32	1.85E-31
01107 Sensor protein PhoQ (*phoQ*)	3.49	1.47	1.62E-04	1.93E-04
01640 Putative phospholipase A1 precursor	2.62	4.82	1.66E-24	3.86E-24
00175 Chemotaxis protein PomA	2.29	7.60	3.21E-29	8.93E-29
02172 Lipopolysaccharide heptosyltransferase 1 (*rfaF*)	3.33	4.75	5.41E-31	1.63E-30
02175 Lipopolysaccharide heptosyltransferase 1 (*rfaF*)	2.37	5.13	2.54E-23	5.61E-23
02179 dTDP-glucose 4,6-dehydratase	1.56	5.80	2.61E-14	4.17E-14
01523 Ribosome maturation factor RimM	4.94	5.22	3.83E-48	2.80E-47
01691 Ribosomal protein S12 methylthiotransferase RimO	2.66	6.76	1.64E-39	7.51E-39
01756 Ribosome maturation factor RimP	4.21	5.85	4.57E-46	2.95E-45
02176 Glucose-1-phosphate thymidylyltransferase 1 (*rmlA*)	1.66	7.52	7.86E-16	1.32E-15
02177 dTDP-4-dehydrorhamnose 3,5-epimerase (*rmlC*)	2.47	6.64	2.64E-31	8.05E-31
02178 dTDP-4-dehydrorhamnose reductase (*rmlD*)	2.92	5.02	1.05E-23	2.35E-23
02102 SPBc2 prophage-derived glycosyltransferase SunS (*sunS*)	4.24	5.25	6.86E-42	3.63E-41
02069 hypothetical protein	3.49	4.63	1.00E-24	2.35E-24
01226 Methyl-accepting chemotaxis protein IV (*mcp4*)	2.97	7.65	1.44E-45	9.08E-45
01311 Methyl-accepting chemotaxis protein IV (*mcp4*)	2.60	3.40	9.01E-11	1.30E-10
01564 Methyl-accepting chemotaxis protein IV (*mcp4*)	2.04	1.61	7.89E-03	8.93E-03
01224 Methyl-accepting chemotaxis protein II	2.78	4.09	4.10E-19	7.75E-19
01655 Methyl-accepting chemotaxis protein II	2.81	5.68	4.54E-38	1.94E-37
01657 Methyl-accepting chemotaxis protein II	1.85	3.47	6.37E-08	8.38E-08
01854 Methyl-accepting chemotaxis protein II	2.03	4.80	4.75E-17	8.40E-17
02058 Methyl-accepting chemotaxis protein II	2.59	4.20	2.19E-15	3.63E-15
00460 Methyl-accepting chemotaxis protein II	2.65	4.25	6.78E-13	1.03E-12
01871 16S/23S rRNA (cytidine-2'-O)-methyltransferase TlyA (*tlyA*)	3.33	5.95	1.54E-29	4.33E-29
00156 Colicin uptake protein TolQ	4.38	7.13	2.61E-53	2.59E-52
00117 TonB-dependent heme receptor A precursor	2.92	2.00	7.66E-06	9.46E-06
01076 Gram-negative bacterial TonB protein (*tonB*)	4.12	4.56	5.67E-27	1.44E-26
01309 TonB dependent receptor	1.79	2.26	1.06E-03	1.24E-03
00918 transport protein TonB (*tonB*)	2.01	4.70	6.52E-14	1.02E-13
00422 Tetrathionate sensor histidine kinase TtrS (*ttrS*)	2.52	4.38	1.66E-18	3.08E-18
02257 Tetrathionate sensor histidine kinase TtrS (*ttrS*)	3.50	1.30	1.01E-03	1.18E-03
02182 N-acetylglucosaminyl-diphospho-decaprenol L-rhamnosyltransferase	4.13	3.80	4.37E-20	8.54E-20
01869 Type II secretion system protein D precursor	2.68	4.81	3.27E-18	6.03E-18
01546 Flagellum site-determining protein YlxH	3.26	6.03	6.50E-28	1.73E-27
02239 Transcriptional regulatory protein ZraR	2.66	4.83	7.90E-17	1.38E-16

Strain LMG10828^T				
00490 Chemotaxis response regulator glutamate methylesterase	1.77	6.85	1.23E-11	3.91E-11
02099 Putative glycosyltransferase EpsJ	2.31	5.12	2.51E-15	1.03E-14
01366 Biopolymer transport protein ExbB (*exbB*)	2.62	9.31	3.04E-29	4.09E-28
01365 Biopolymer transport protein ExbD (*exbD*)	2.65	7.70	1.01E-27	1.17E-26
01208 Sensor protein FixL	1.67	3.15	2.78E-05	5.41E-05
01958 Flagellar assembly protein H	2.13	5.87	8.21E-17	3.79E-16
00565 Flagellin	-1.67	9.33	9.03E-12	2.9E-11
01960 Flagellar M-ring protein	2.26	6.46	1.63E-19	9.15E-19
00972 flagellar motor switch protein	1.68	7.64	1.07E-12	3.71E-12
01961 Flagellar basal body rod protein FlgB	1.64	7.54	4.05E-12	1.35E-11
01935 Flagellar biosynthesis protein FlhA	1.62	5.29	1.24E-07	2.97E-07
01959 Flagellar motor switch protein FliG	2.03	6.46	2.28E-16	1E-15
00683 Flagellar biosynthetic protein FliP precursor	3.01	6.87	7.93E-32	1.52E-30
00026 Flagellar biosynthetic protein FliQ	1.58	7.32	9.72E-09	2.53E-08
01940 flagellar biosynthesis protein FliR	1.75	5.88	7.05E-11	2.11E-10
00673 Gram-negative bacterial TonB protein (*tonB*)	1.78	5.47	9.31E-10	2.57E-09
00740 Hemolysin transporter protein ShlB precursor	1.96	2.90	5.16E-06	1.09E-05
00452 Methyl-accepting chemotaxis protein IV	3.22	4.85	3.03E-20	1.8E-19
00353 Methyl-accepting chemotaxis protein IV	2.15	6.91	7.89E-20	4.51E-19
00331 Methyl-accepting chemotaxis protein 4	2.91	6.13	1.41E-16	6.38E-16
00834 Methyl-accepting chemotaxis protein II	1.95	5.65	1.38E-12	4.73E-12
02234 Methyl-accepting chemotaxis protein II	1.55	4.28	1.12E-07	2.69E-07
00802 putative peptidoglycan biosynthesis protein MurJ (*mviN*)	2.02	3.90	1.87E-10	5.42E-10
02096 O-Antigen ligase	1.53	4.35	4.82E-06	1.01E-05
02078 Transcriptional regulatory protein PhoP (*phoP*)	1.51	5.41	8.03E-06	1.66E-05
02038 Virulence transcriptional regulatory protein PhoP (*phoP*)	1.60	7.43	2.19E-06	4.71E-06
00642 Sensor protein PhoQ (*phoQ*)	1.60	2.67	0.00039	0.000663
02180 cryptic beta-D-galactosidase subunit beta	1.74	6.35	1.49E-10	4.35E-10
00164 TonB-dependent heme receptor A precursor	1.85	2.12	0.0007	0.001159
01740 Transport protein TonB (*tonB*)	1.84	7.28	5.13E-07	1.16E-06
02079 Tetrathionate sensor histidine kinase TtrS (*ttrS*)	1.53	1.98	0.010922	0.015705
02212 Urease accessory protein UreD	1.70	2.83	8.34E-05	0.000155
02209 Urease accessory protein UreE	1.55	4.00	1.96E-05	3.88E-05
02208 Urease accessory protein UreF	2.06	2.70	1.88E-05	3.74E-05
01307 Sensor protein ZraS	1.68	5.92	1.39E-05	2.81E-05
Strain 31				
00607 Chemotaxis protein methyltransferase (*cheR*)	1.60	7.37	1.95E-07	4.22E-07
00609 Chemotaxis response regulator glutamate methylesterase	2.76	7.00	1.02E-19	1.03E-18
00608 Chemoreceptor glutamine deamidase CheD (*cheD*)	2.06	6.65	2.25E-10	6.46E-10
01893 CheW-like domain protein (*cheW*)	2.05	3.20	2.54E-06	4.99E-06
01607 Chemotaxis protein CheY (*cheY*)	2.04	8.49	1.82E-11	5.87E-11

01466 Putative glycosyltransferase EpsD	1.91	4.26	1.28E-07	2.81E-07
00986 Biopolymer transport protein ExbB (*exbB*)	2.68	9.78	2.11E-11	6.76E-11
00233 Biopolymer transport protein ExbD (*exbD*)	2.09	5.31	7.58E-10	2.06E-09
00987 Biopolymer transport protein ExbD (*exbD*)	2.76	8.20	3.95E-11	1.22E-10
01606 flagellar motor switch protein	2.62	7.55	6.07E-20	6.39E-19
01946 flagellar assembly protein H	2.50	6.49	2.39E-19	2.28E-18
01731 Flagellar L-ring protein precursor	1.92	6.76	1.03E-10	3.07E-10
01948 Flagellar M-ring protein	2.49	6.73	3.21E-20	3.68E-19
00550 Flagellin	-1.82	9.61	5.96E-07	1.24E-06
01949 Flagellar basal body rod protein FlgB	1.84	7.71	2.92E-11	9.18E-11
01923 Flagellar biosynthesis protein FlhA	2.40	6.65	1.15E-16	7.09E-16
01332 Flagellar biosynthetic protein FlhB	1.83	5.49	2.92E-09	7.44E-09
01934 Flagellar biosynthesis protein FlhF	2.61	6.39	1.26E-18	1.08E-17
01947 Flagellar motor switch protein FliG	2.41	6.87	5.47E-19	4.97E-18
01929 Flagellar hook-length control protein FliK	1.63	7.08	5.28E-07	1.1E-06
01723 Flagellar motor switch protein FliM	1.52	5.07	1.09E-06	2.2E-06
00445 Flagellar biosynthetic protein FliP precursor	2.55	7.15	4.52E-15	2.2E-14
01726 Flagellar biosynthetic protein FliQ	1.82	7.18	6.39E-09	1.57E-08
01928 flagellar biosynthesis protein FliR	2.23	5.80	1.25E-12	4.5E-12
01914 Methyl-accepting chemotaxis protein IV (*mcp4*)	1.91	3.40	3.56E-05	6.29E-05
00106 Methyl-accepting chemotaxis protein 4 (*mcp4*)	2.19	5.55	7.53E-12	2.52E-11
02131 Methyl-accepting chemotaxis protein II	2.14	4.24	2.83E-09	7.22E-09
00007 Methyl-accepting chemotaxis protein IV (*mcp4*)	2.17	5.08	2.43E-11	7.76E-11
00214 Motility protein B	2.15	6.89	4.95E-15	2.38E-14
00343 putative peptidoglycan biosynthesis protein MurJ (*mviN*)	2.84	4.56	1.96E-14	8.72E-14
00132 Outer membrane porin F precursor (*oprF; cadF*)	-1.53	11.68	7.01E-05	0.00012
00815 Outer membrane porin F precursor (*oprF; cadF*)	1.58	6.56	8.77E-07	1.79E-06
00554 Response regulator MprA (*mprA*)	1.82	3.47	1.31E-05	2.39E-05
01378 Response regulator MprA (*mprA*)	1.89	7.55	6.23E-06	1.17E-05
02043 cryptic beta-D-galactosidase subunit beta	2.69	6.71	3.81E-17	2.47E-16
01440 TlyA (*tlyA*)	2.03	6.72	1.99E-09	5.17E-09
00455 Gram-negative bacterial TonB protein (*tonB*)	2.25	5.31	4.23E-10	1.18E-09
01819 Transport protein TonB (*tonB*)	1.63	8.32	5.18E-06	9.85E-06
01043 Tetrathionate sensor histidine kinase TtrS (*ttrS*)	1.60	3.81	7.94E-06	1.48E-05
02069 Urease subunit alpha	1.57	2.61	0.002277	0.00337
02067 Urease accessory protein UreE	1.92	3.73	5.56E-07	1.16E-06
02066 Urease accessory protein UreF	2.44	2.49	8.54E-06	1.58E-05
02065 Urease accessory protein UreG	1.95	3.69	8.4E-07	1.72E-06
00887 Sensor protein ZraS	2.04	3.33	8.94E-06	1.65E-05

Supplementary Table 3. *A. butzleri* DEGs after 30 minutes of contact with host cells. The table shows logFC (< -1.5, > 1.5) and *p value* (< 0.05) of differentially expressed genes linked to the currently considered putative virulence genes. The column under the strain name shows the protein name of the DEGs and the relative locus tag. The name of the genes indicated in the main text is indicated in brackets after the name of the protein.

**LMG 11119**	**logFC**	**p value**	**31**	**logFC**	**p value**	**LMG 10828^T**	**logFC**	**p value**
00920 Biopolymer transport protein ExbB (*exbB*)	2.13	7.19E-20	00234 hypothetical protein	1.84	5.28E-04	00201 Acetaldehyde dehydrogenase 2	2.58	5.55E-21
01311 Methyl-accepting chemotaxis protein IV (*mcp4*)	3.10	3.49E-19	00235 Acetaldehyde dehydrogenase 2	4.01	2.85E-08	01089 Cytochrome c-type protein SHP precursor	2.35	7.13E-09
00918 Transport protein TonB (*tonB*)	1.77	1.38E-16	00363 Cytochrome c	3.37	5.18E-07	00640 hypothetical protein	-1.55	1.78E-04
00082 NADH-quinone oxidoreductase subunit I	1.54	1.57E-15	00744 hypothetical protein	-3.86	9.33E-05	00624 hypothetical protein	-2.20	1.01E-11
00131 Putative electron transport protein YccM	1.61	2.43E-10	00745 Phosphate import ATP-binding protein PstB	-3.35	1.23E-03	01903 hypothetical protein	-1.76	8.27E-04
00143 hypothetical protein	1.50	5.06E-14	00747 Phosphate transport system permease protein PstC	-1.89	9.43E-04	00325 Inner membrane protein YjcH (*yjcH*)	3.15	1.50E-32
00145 30S ribosomal protein S15	1.73	1.74E-19	00750 Phosphate-binding protein PstS precursor	-2.59	6.19E-06	00324 Cation/acetate symporter ActP (*actP*)	2.90	1.12E-20
00153 Acetaldehyde dehydrogenase 2	-2.46	3.31E-39	01598 Integral membrane protein TerC family protein	-1.78	1.45E-03	00315 Acetate kinase (*ackA*)	2.21	9.31E-17
00183 hypothetical protein	1.51	1.08E-12	00112 Inner membrane protein YjcH (*yjcH*)	2.39	2.82E-06	00316 Phosphate acetyltransferase (*pta*)	2.16	1.03E-13
00352 Antibiotic biosynthesis monooxygenase	1.66	7.29E-09	00113 Cation/acetate symporter ActP (*actP*)	1.81	3.29E-04	00187 Putative regulator of ribonuclease activity	2.46	3.04E-12
00475 Helix-turn-helix domain protein	1.75	1.48E-16	00265 Cytochrome c-type protein NrfH	5.13	2.54E-12	02298 Transglutaminase-like superfamily protein	6.80	1.23E-11
00610 hypothetical protein	1.73	2.25E-03	00371 hypothetical protein	2.28	6.20E-07	02076 hypothetical protein	-6.91	7.68E-06
00613 RNA recognition motif. (a.k.a. RRM, RBD, or RNP domain)	1.65	9.43E-20	00411 hypothetical protein	1.52	3.27E-04	01448 hypothetical protein	-5.64	9.09E-04
00693 fec operon regulator FecR	1.60	3.97E-06	00434 OstA-like protein	-1.94	2.64E-04	02291 Formate hydrogenlyase complex iron-sulfur subunit	6.70	2.21E-20
00881 hypothetical protein	1.88	1.22E-16	00550 Flagellin	2.13	2.11E-05	01172 hypothetical protein	1.51	1.42E-12
00992 CheW-like domain protein	1.62	4.28E-06	00854 Cytochrome c-type protein SHP precursor	5.04	9.18E-19	02292 Receptor family ligand binding region	6.28	1.66E-06
01028 Aerotaxis receptor	1.87	1.94E-10	01106 Maf-like protein	-9.38	5.48E-07	02290 Twin-argninine leader-binding protein DmsD	6.32	5.97E-08

01289 Cysteine desulfurase	2.38	5.25E-34	01250 ATP-dependent RNA helicase RhlE	3.87	4.67E-10	00588 Cytochrome bd-I ubiquinol oxidase subunit 1	-1.96	2.47E-04
01290 NifU-like protein	2.29	2.13E-35	01713 hypothetical protein	-1.60	3.51E-04	00980 hypothetical protein	-5.98	2.06E-04
01409 30S ribosomal protein S21	2.28	1.22E-25	01795 2-methylcitrate synthase	1.96	2.23E-05	02293 Sensor protein FixL	5.77	8.64E-07
01436 recombinase A	1.72	1.41E-17	02039 hypothetical protein	-8.80	2.21E-06	02295 Natural resistance-associated macrophage protein	5.53	4.85E-06
01666 hypothetical protein	-1.84	1.94E-05	02141 Copper chaperone CopZ	-8.11	3.24E-05	01598 Periplasmic serine endoprotease DegP precursor	2.11	8.44E-18
01668 Phosphatidate cytidylyltransferase	-1.61	1.10E-06	02153 Cell wall-associated hydrolase	2.32	1.29E-05	00100 Aconitate hydratase	2.20	9.91E-12
01778 2-aminoadipate transaminase	1.86	2.47E-08	00025 YceI-like domain protein	4.59	1.75E-15	00264 Macrolide export ATP-binding/permease protein MacB (*macB*)	6.70	3.77E-32
01784 SnoaL-like polyketide cyclase	1.84	1.04E-23	00172 Macrolide export ATP-binding/permease protein MacB (*macB*)	4.92	1.63E-08	00982 Helix-turn-helix domain protein	-7.14	2.46E-06
01849 Hydrogenase expression/formation protein HypD	1.56	1.43E-08	00173 Lipoprotein-releasing system ATP-binding protein LolD	4.17	5.63E-04	01339 HicB family protein	-1.66	1.29E-09
01850 Hydrogenase isoenzymes formation protein HypC	1.70	2.01E-10	00231 D-cysteine desulfhydrase	-6.75	9.68E-04	02294 Transcriptional regulatory protein ZraR	5.10	9.30E-06
01851 Hydrogenase/urease nickel incorporation protein HypB	1.88	2.50E-20	00266 Cytochrome c-552 precursor	4.48	2.42E-13	01422 Modification methylase DpnIIA	-6.22	4.93E-05
01860 hypothetical protein	1.58	4.39E-17	00374 hypothetical protein	2.36	1.43E-04	01765 Colicin I receptor precursor (*cirA; irgA*)	1.86	4.81E-03
01866 hypothetical protein	-2.11	7.29E-04	00522 hypothetical protein	2.33	2.71E-04	00727 hypothetical protein	-2.60	3.60E-12
02019 hypothetical protein	1.61	1.27E-11	00555 Outer membrane porin F precursor (*oprF; cadF*)	2.21	6.90E-05	00101 3-methylitaconate isomerase	2.56	6.93E-10
02032 putative HTH-type transcriptional regulator YxaF	1.69	4.03E-15	00568 3,4-dihydroxy-2-butanone 4-phosphate synthase	7.42	2.14E-06	00262 hypothetical protein	5.47	3.05E-14
02035 DNA polymerase IV	1.51	2.34E-11	00596 Ribosome-associated factor Y	1.82	2.88E-05	00260 hypothetical protein	4.76	9.53E-17
02079 Radical SAM superfamily protein	1.84	4.21E-13	00645 Divalent-cation tolerance protein CutA	-8.33	1.28E-05	00586 hypothetical protein	-2.66	1.73E-03
02184 YciI-like protein	1.80	3.24E-23	00823 hypothetical protein	3.27	6.79E-08	01338 hypothetical protein	-1.67	1.65E-10
02255 hypothetical protein	-5.42	1.17E-04	00894 hypothetical protein	-8.87	3.03E-06	02077 hypothetical protein	-6.40	3.87E-05

00777 Flagellin	-1.55	1.20E-17	00902 Imelysin	-2.11	7.26E-04	02289 hypothetical protein	5.50	2.26E-30
00277 Inner membrane protein YjcH (*yjcH*)	1.73	1.68E-05	01056 hypothetical protein	2.83	3.93E-04	00998 hypothetical protein	-2.18	2.39E-05
			01077 hypothetical protein	8.95	6.48E-07	00263 Lipoprotein-releasing system ATP-binding protein LolD	6.55	2.88E-21
00239 Helix-turn-helix domain protein	2.00	1.91E-02	01105 Phage integrase family protein	6.19	3.23E-04	01762 Catecholate siderophore receptor Fiu precursor (*fiu*)	2.49	5.40E-06
00240 Quinohemoprotein amine dehydrogenase A, alpha subunit, haem binding	1.87	9.18E-04	01313 Methyl-accepting chemotaxis protein II	-1.69	2.42E-04	02310 hypothetical protein	3.46	5.95E-07
00242 Quinohemoprotein amine dehydrogenase subunit gamma	2.18	9.74E-04	01347 tRNA-Glu(ttc)	-8.33	1.67E-05	00778 Cytochrome c	2.56	2.87E-23
00274 DNA polymerase III PolC-type	1.55	6.08E-04	01401 hypothetical protein	-8.29	2.87E-05	01458 hypothetical protein	-6.17	5.67E-05
00470 transfer-messenger RNA, SsrA	-2.15	7.48E-19	01444 Methyl-accepting chemotaxis protein IV (*mcp4*)	2.67	7.13E-04	00558 Outer membrane porin F precursor (*oprF; cadF*)	1.68	3.05E-08
00490 Helix-turn-helix domain protein	-1.56	1.06E-02	01461 Putative teichuronic acid biosynthesis glycosyltransferase TuaH	7.53	1.09E-04	00983 hypothetical protein	-3.29	4.61E-03
00557 Septum site-determining protein MinD	1.54	1.76E-18	01467 Peptidoglycan O-acetyltransferase	5.97	5.96E-05	00471 Outer membrane lipoprotein Blc precursor	-1.99	6.73E-16
00692 putative RNA polymerase sigma factor FecI	1.92	2.31E-06	01497 Von Willebrand factor type A domain protein	2.59	4.03E-04	02287 Formate dehydrogenase H	4.46	1.65E-21
00694 Ferric-pseudobactin BN7/BN8 receptor precursor (*pupB*)	1.53	3.62E-03	01582 Flagellar filament 33 kDa core protein	3.03	1.11E-10	01454 hypothetical protein	-5.29	2.98E-03
00829 hypothetical protein	-2.28	3.93E-03	01610 hypothetical protein	-8.08	2.92E-05	01597 Transcriptional regulatory protein BaeR	1.52	2.37E-07
00935 hypothetical protein	2.22	2.80E-13	01794 Methylisocitrate lyase	1.83	1.27E-04	00261 hypothetical protein	4.06	1.76E-08
00940 Catecholate siderophore receptor Fiu precursor (*fiu*)	1.84	1.54E-05	01820 Biopolymer transport protein ExbD (*exbD*)	-1.75	2.52E-04	00989 hypothetical protein	-5.68	6.17E-04
01101 Cadherin domain protein	-1.53	5.75E-07	01903 Transposase DDE domain protein	-7.07	4.86E-04	00555 Oxygen regulatory protein NreC	1.58	1.40E-04
01210 Ferrichrome receptor FcuA precursor (*fcuA*)	1.95	3.74E-05						

01568 bifunctional aldehyde dehydrogenase/enoyl-CoA hydratase	1.70	2.01E-14
01570 (3S)-malyl-CoA thioesterase	1.83	3.85E-21
01602 Cytochrome c	-3.27	2.51E-63
01665 zinc-responsive transcriptional regulator	-1.67	5.58E-07
01667 CDP-alcohol phosphatidyltransferase	-1.84	3.30E-08
01795 Iron uptake protein A1 precursor (*fur*)	1.73	8.87E-20
01919 SkfA peptide export ATP-binding protein SkfE	2.48	1.78E-18
01920 hypothetical protein	1.58	1.30E-04
01921 Ferrous iron transport protein A (*feoA*)	1.67	2.12E-05
02252 Cell wall-associated hydrolase	-2.68	1.09E-06
02009 UDP-N-acetyl-D-glucosamine 6-dehydrogenase	7.86	1.19E-08
02082 Transcriptional activator protein CopR	-6.86	7.44E-04
02163 Methyl-accepting chemotaxis protein I	2.65	3.83E-07
01483 Tyrosine recombinase XerD	-2.09	1.54E-04
00616 Putative NAD(P)H nitroreductase YfkO	-1.56	5.98E-12
00011 Zinc-responsive transcriptional regulator	-2.32	7.51E-09
00992 hypothetical protein	-5.23	3.06E-03
00472 protoporphyrinogen oxidase	-1.86	1.34E-14
00619 Putative monooxygenase YcnE	-2.41	4.97E-09
00620 Modulator of drug activity B	-2.07	4.92E-15
00618 NADPH dehydrogenase	-2.28	5.58E-20
00284 Cobalt-zinc-cadmium resistance protein CzcB	-2.28	9.22E-06
02150 hypothetical protein	-1.80	1.21E-03
02271 hypothetical protein	-6.73	4.09E-06
00617 EamA-like transporter family protein	-2.21	9.32E-19
00425 Aerotaxis receptor	-1.96	2.26E-12
00991 hypothetical protein	-5.18	4.60E-03
00630 Sodium:sulfate symporter transmembrane region	-1.56	9.45E-04
00259 hypothetical protein	3.75	2.18E-06
01510 hypothetical protein	-6.67	4.80E-06
00631 General stress protein 14	-1.64	1.20E-06
00015 1-acyl-sn-glycerol-3-phosphate acyltransferase	-1.86	5.12E-08
00012 hypothetical protein	-2.69	6.06E-09
01485 hypothetical protein	-6.53	2.20E-05

	00014 Phosphatidate cytidylyltransferase	-1.77	1.80E-13
	01487 hypothetical protein	-6.14	9.38E-05
	00573 Hemin transport system permease protein HmuU	-2.39	4.28E-03
	01488 TraM recognition site of TraD and TraG	-5.67	8.88E-04
	00426 Methyl-accepting chemotaxis protein IV (*mcp4*)	-1.97	3.73E-10
	01596 Sensor protein BasS	2.14	2.93E-13
	02034 Tyrosine recombinase XerC	-2.09	1.44E-04
	02288 Putative formate dehydrogenase	3.45	5.83E-20
	01087 Phosphoethanolamine transferase EptA	3.22	4.59E-16
	01611 hypothetical protein	-1.53	6.49E-04
	01307 Sensor protein ZraS	-1.55	2.79E-04
	01088 PAP2 superfamily protein	3.59	7.83E-23
	01486 hypothetical protein	-5.60	9.62E-04
	01141 Lactate utilization protein C	1.63	2.99E-06
	01008 Prophage CP4-57 integrase	-1.52	1.74E-07
	01887 hypothetical protein	-1.54	1.59E-03
	01603 Prophage CP4-57 integrase	-1.97	2.12E-09
	01609 hypothetical protein	-2.30	4.47E-03
	02100 Putative teichuronic acid biosynthesis glycosyltransferase TuaC	-1.62	2.48E-06
	00961 tRNA-Glu(ttc)	-8.90	2.96E-14
	00290 Quinohemoprotein amine dehydrogenase subunit gamma	-6.09	1.05E-04
	01610 hypothetical protein	-3.91	1.99E-04

02237 Regulatory protein PchR	-1.88	7.55E-10
00645 Chaperone protein DnaJ	-2.77	6.36E-04
01613 hypothetical protein	-1.61	3.85E-04
01607 hypothetical protein	-5.62	1.65E-03
01608 hypothetical protein	-2.50	3.98E-03
01138 Glycolate permease GlcA	1.62	1.43E-07
02238 Ferrichrome receptor FcuA precursor (*fcuA*)	-3.07	5.70E-04

Supplementary Table 4. *A. butzleri* DEGs after 90 minutes of contact with host cells. The table shows logFC (< -1.5, > 1.5) and *p value* (< 0.05) of differentially expressed genes linked to the currently considered putative virulence genes. The column under the strain name shows the protein name of the DEGs and the relative locus tag. The name of the genes indicated in the main text is indicated in brackets after the name of the protein.

LMG 11119	logFC	p value	31	logFC	p value	LMG 10828T	logFC	p value
00920 Biopolymer transport protein ExbB (*exbB*)	3.13	8.2E-50	00164 Ribosomal RNA large subunit methyltransferase H	-1.92	3.98E-03	00201 Acetaldehyde dehydrogenase 2	2.70	9E-07
00918 Transport protein TonB (*tonB*)	2.72	1.6E-39	00235 Acetaldehyde dehydrogenase 2	3.61	5.06E-07	01089 Cytochrome c-type protein SHP precursor	2.22	2E-05
00082 NADH-quinone oxidoreductase subunit I	2.12	2.4E-31	00334 hypothetical protein	-10.24	9.77E-09	01900 Heat shock protein GrpE	-1.51	2E-05
00145 30S ribosomal protein S15	2.72	2.6E-46	00363 Cytochrome C	3.15	2.06E-06	01901 Chaperone protein DnaK	-1.82	3E-08
00153 Acetaldehyde dehydrogenase 2	-2.72	2.1E-35	00399 hypothetical protein	-8.62	7.13E-05	00624 hypothetical protein	-3.25	3E-14
00610 hypothetical protein	2.40	2.0E-04	00435 Putative GTP-binding protein EngB	-1.80	2.56E-03	01171 Cation efflux system protein CusA	1.92	4E-03
00693 Fec operon regulator FecR	2.51	5.2E-18	00447 hypothetical protein	-8.10	1.45E-04	01902 Membrane-bound lytic murein transglycosylase A precursor	-2.31	6E-13
01289 Cysteine desulfurase	1.73	1.7E-14	00466 50S ribosomal protein L36	-1.56	1.75E-04	01009 tRNA-Leu(caa)	-2.03	6E-09
01290 NifU-like protein	1.87	2.4E-19	00470 DNA-directed RNA polymerase subunit alpha	-1.51	5.05E-04	00906 Hydrogenase 2 maturation protease	-1.51	3E-03
01409 30S ribosomal protein S21	2.76	4.8E-42	00471 50S ribosomal protein L17	-1.56	8.91E-05	01318 Chaperone protein HtpG	-2.23	3E-10

01666 hypothetical protein	-1.82	1.1E-05	00520 Formyltetrahydrofolate deformylase	-1.62	1.53E-03	00904 Quinone-reactive Ni/Fe-hydrogenase large chain	-2.80	1E-11
01668 Phosphatidate cytidylyltransferase	-1.60	4.5E-07	00745 Phosphate import ATP-binding protein PstB	-3.11	2.81E-03	00905 Quinone-reactive Ni/Fe-hydrogenase B-type cytochrome subunit	-2.05	4E-07
01784 SnoaL-like polyketide cyclase	2.06	5.2E-28	00747 Phosphate transport system permease protein PstC	-2.33	2.22E-04	00903 Quinone-reactive Ni/Fe-hydrogenase small chain precursor	-3.15	3E-18
01850 Hydrogenase isoenzymes formation protein HypC	1.57	3.5E-07	00750 Phosphate-binding protein PstS precursor	-1.64	2.04E-03	00325 Inner membrane protein YjcH (*yjcH*)	3.53	4E-28
01860 hypothetical protein	1.61	6.2E-14	00960 tRNA modification GTPase MnmE	-1.56	4.29E-03	00324 Cation/acetate symporter ActP (*actP*)	3.80	2E-28
02184 YciI-like protein	1.89	5.3E-22	00972 GTPase Obg	-1.61	1.30E-03	00315 Acetate kinase (*ackA*)	2.41	1E-14
00777 Flagellin	-1.91	1.2E-24	00974 Methionyl-tRNA formyltransferase	-1.68	5.65E-03	00316 Phosphate acetyltransferase (*pta*)	2.53	2E-14
00277 Inner membrane protein YjcH (*yjcH*)	2.82	1.6E-10	00994 Aminodeoxyfutalosine nucleosidase	-2.20	1.98E-04	01287 hypothetical protein	1.50	2E-06
00274 DNA polymerase III PolC-type	2.40	6.5E-06	01195 ribosome-binding factor A	-1.53	2.52E-04	00187 Putative regulator of ribonuclease activity	3.00	2E-12
00470 transfer-messenger RNA, SsrA	-2.11	2.0E-26	01300 hypothetical protein	-8.32	1.04E-04	02298 Transglutaminase-like superfamily protein	5.13	5E-06
00490 Helix-turn-helix domain protein	-1.64	4.7E-03	01425 tRNA-Met(cat)	-1.81	1.65E-05	01290 hypothetical protein	1.95	2E-07
00557 Septum site-determining protein MinD	1.58	7.1E-16	01455 Diacylglycerol kinase	-10.48	3.58E-10	02291 formate hydrogenlyase complex iron-sulfur subunit	5.81	5E-10
00692 putative RNA polymerase sigma factor FecI	2.84	3.5E-20	01511 hypothetical protein	-9.65	1.09E-06	02290 twin-argninine leader-binding protein DmsD	5.17	1E-03
00694 Ferric-pseudobactin BN7/BN8 receptor precursor (*pupB*)	3.36	7.8E-17	01598 Integral membrane protein TerC family protein	-2.36	6.65E-06	00587 hypothetical protein	-7.21	6E-07
00935 hypothetical protein	3.19	4.3E-34	01889 Translation initiation factor IF-1	-1.91	4.62E-04	00638 putative RNA polymerase sigma factor FecI	1.53	8E-04
00940 Catecholate siderophore receptor Fiu precursor (*fiu*)	3.77	3.2E-18	01956 Ribosome maturation factor RimM	-1.94	1.38E-03	01771 Carbon starvation protein A	1.81	3E-09
01210 Ferrichrome receptor FcuA precursor (*fcuA*)	3.65	1.2E-17	01958 30S ribosomal protein S16	-2.01	1.53E-05	02293 Sensor protein FixL	5.03	7E-04
01602 Cytochrome c	-4.41	6.1E-62	01961 3-deoxy-D-manno-octulosonic acid transferase	-2.00	1.75E-03	02143 Iron-regulated protein A precursor	1.57	2E-04
01665 zinc-responsive transcriptional regulator	-1.85	1.6E-09	02033 50S ribosomal protein L25	-1.79	5.04E-05	01357 Amino-acid carrier protein AlsT	1.75	1E-04

01667 CDP-alcohol phosphatidyltransferase	-1.78	2.5E-09	02099 tRNA-Phe(gaa)	-1.54	1.26E-04	02295 Natural resistance-associated macrophage protein	4.27	8E-04
01795 Iron uptake protein A1 precursor (*fur*)	2.56	6.2E-39	02112 Bifunctional DNA-directed RNA polymerase subunit beta-beta'	-1.52	1.28E-03	00767 hypothetical protein	-1.55	7E-06
01919 SkfA peptide export ATP-binding protein SkfE	4.02	2.7E-59	00009 UDP-2,3-diacylglucosamine hydrolase	-8.02	1.95E-04	02242 Helix-hairpin-helix motif protein	1.96	9E-09
01920 hypothetical protein	2.87	4.8E-14	00025 YceI-like domain protein	4.35	2.12E-14	01460 hypothetical protein	2.41	4E-04
01921 Ferrous iron transport protein A (*feoA*)	3.23	6.0E-16	00030 hypothetical protein	4.40	1.21E-03	01598 Periplasmic serine endoprotease DegP precursor	2.32	2E-11
02252 Cell wall-associated hydrolase	-2.90	1.2E-09	00038 hypothetical protein	-6.42	4.83E-03	00264 Macrolide export ATP-binding/permease protein MacB	4.99	9E-11
00919 Biopolymer transport protein ExbD (*exbD*)	2.29	2.5E-24	00075 hypothetical protein	-2.16	1.59E-03	00982 Helix-turn-helix domain protein	-7.15	3E-06
00943 Colicin I receptor precursor (*cirA; irgA*)	2.40	1.8E-08	00089 hypothetical protein	-7.39	7.69E-04	01437 hypothetical protein	3.70	3E-05
00042 hypothetical protein	1.61	4.9E-16	00090 hypothetical protein	-8.00	2.44E-04	01339 HicB family protein	-1.85	1E-08
00067 hypothetical protein	1.68	5.1E-18	00112 Inner membrane protein YjcH (*yjcH*)	2.39	3.46E-06	01770 hypothetical protein	1.62	6E-05
00068 50S ribosomal protein L32	1.73	2.7E-20	00113 Cation/acetate symporter ActP (*actP*)	1.93	8.99E-06	01765 Colicin I receptor precursor (*cirA; irgA*)	4.29	1E-09
00083 NADH-quinone oxidoreductase subunit H	1.92	4.9E-24	00117 DNA polymerase III PolC-type	-6.42	5.04E-03	00727 hypothetical protein	-2.44	5E-10
00084 NADH-quinone oxidoreductase subunit 3	1.77	8.6E-21	00146 hypothetical protein	-7.20	1.18E-03	00262 hypothetical protein	4.15	1E-05
00089 NADH-quinone oxidoreductase subunit 6	1.54	3.9E-14	00147 Quinohemoprotein amine dehydrogenase subunit gamma	-6.76	3.07E-03	00260 hypothetical protein	2.97	4E-06
00109 Regulatory protein PchR	1.62	7.7E-09	00156 Blue-light-activated protein	6.23	1.14E-04	00977 Nitric oxide reductase subunit B	-1.77	3E-05
00121 Cytochrome c biogenesis protein CcsA	-1.68	6.3E-05	00157 Response regulator MprA (*mprA*)	8.16	1.48E-05	01758 Fumarate hydratase class II	2.20	5E-08
00123 Cytochrome c-type protein NrfH	-1.67	4.3E-15	00172 Macrolide export ATP-binding/permease protein MacB (*macB*)	8.37	1.06E-46	00535 Cytochrome c-type protein TorY	-1.51	1E-04
00236 Cobalt-zinc-cadmium resistance protein CzcB	-2.09	9.2E-17	00173 Lipoprotein-releasing system ATP-binding protein LolD	8.08	4.65E-34	01289 hypothetical protein	2.01	1E-07
00237 Outer membrane efflux protein	-2.06	5.8E-19	00174 hypothetical protein	6.81	1.69E-23	02041 Outer membrane efflux protein	2.17	4E-03
00238 Fatty acid metabolism regulator protein	-1.70	9.8E-17	00175 hypothetical protein	5.98	1.61E-13	00163 Regulatory protein PchR	2.52	9E-05
00260 Ribose-phosphate pyrophosphokinase	1.93	2.0E-27	00176 hypothetical protein	5.99	3.27E-25	01338 hypothetical protein	-1.65	8E-07
00287 tRNA-Val(tac)	1.53	1.8E-13	00177 hypothetical protein	4.79	1.61E-04	02289 hypothetical protein	5.12	1E-15

00290 tRNA-Asp(gtc)	1.54	6.4E-17	00189 Ubiquinone/menaquinone biosynthesis C-methyltransferase UbiE	-2.27	1.45E-03	02045 FhuE receptor precursor	3.23	2E-08
00291 tRNA-Val(tac)	1.65	7.8E-12	00224 Small-conductance mechanosensitive channel	-1.74	5.09E-03	00998 hypothetical protein	-2.04	1E-04
00309 hypothetical protein	2.00	1.2E-11	00265 Cytochrome c-type protein NrfH	5.64	1.60E-25	01444 hypothetical protein	3.24	6E-06
00357 fec operon regulator FecR	1.78	1.4E-05	00266 Cytochrome c-552 precursor	4.41	1.54E-16	01331 YceI-like domain protein	2.81	1E-08
00381 ATP synthase epsilon chain	1.97	9.0E-22	00347 Fluoroacetyl-CoA thioesterase	1.54	2.19E-04	02048 hypothetical protein	2.25	2E-07
00382 ATP synthase subunit beta	1.69	1.4E-15	00352 Thiol-disulfide oxidoreductase ResA	-1.76	3.47E-03	00857 Dihydrolipoyl dehydrogenase	1.65	7E-07
00497 hypothetical protein	1.58	1.5E-12	00371 hypothetical protein	2.93	3.05E-11	02042 hypothetical protein	2.70	5E-05
00504 tRNA-His(gtg)	1.80	1.7E-17	00376 hypothetical protein	-1.60	4.88E-03	00263 Lipoprotein-releasing system ATP-binding protein LolD	4.04	4E-06
00505 tRNA-Pro(tgg)	1.63	9.8E-10	00404 hypothetical protein	5.65	1.76E-10	01648 Succinate-semialdehyde dehydrogenase [NADP(+)]	1.53	1E-06
00521 50S ribosomal protein L31	1.96	2.7E-24	00405 hypothetical protein	-6.53	4.99E-03	02165 hypothetical protein	2.05	6E-08
00525 tRNA-Arg(tcg)	1.96	1.3E-17	00409 Lumazine-binding domain protein	-10.44	9.22E-10	01762 Catecholate siderophore receptor Fiu precursor (*fiu*)	4.45	5E-18
00538 Spermidine synthase	1.58	1.8E-16	00411 hypothetical protein	1.88	1.59E-05	01766 Ferri-bacillibactin esterase BesA (*iroE*)	2.12	2E-04
00544 hypothetical protein	-1.60	8.7E-14	00413 hypothetical protein	-9.56	2.03E-06	01761 Transcriptional regulatory protein WalR (*walR*)	1.74	9E-04
00564 Putative oxidoreductase CatD	1.83	3.8E-24	00458 Putative F0F1-ATPase subunit (ATPase_gene1)	-1.89	4.48E-05	02310 hypothetical protein	2.85	2E-04
00589 Peptide chain release factor 1	1.59	3.2E-17	00474 Cupin domain protein	2.21	1.29E-05	02049 hypothetical protein	2.27	6E-06
00590 30S ribosomal protein S20	1.53	1.6E-16	00522 hypothetical protein	2.09	1.15E-03	00778 Cytochrome c	2.67	1E-08
00598 50S ribosomal protein L20	2.28	1.3E-30	00525 hypothetical protein	-1.93	5.38E-03	00994 hypothetical protein	-5.38	2E-03
00599 50S ribosomal protein L35	2.17	1.2E-26	00527 Signal transduction histidine-protein kinase/phosphatase MprB	-8.00	1.99E-04	00558 Outer membrane porin F precursor (*oprF; cadF*)	1.83	1E-07
00607 Cation efflux system protein CusA	1.99	4.2E-04	00542 Hemin transport system permease protein HmuU	-7.40	7.12E-04	01341 ECF RNA polymerase sigma factor SigE	1.99	5E-03
00608 Cation efflux system protein CusB precursor	1.69	8.0E-03	00547 Sensor protein RstB	-2.23	3.19E-03	02287 Formate dehydrogenase H	4.42	1E-12
00609 Outer membrane efflux protein	1.84	4.4E-03	00550 Flagellin	1.79	8.85E-05	00922 hypothetical protein	-1.60	4E-04
00623 Ribosomal large subunit pseudouridine synthase B	1.57	5.9E-14	00555 Outer membrane porin F precursor (*oprF; cadF*)	1.91	1.93E-03	01597 Transcriptional regulatory protein BaeR	2.10	5E-08

00652 Ribonucleoside-diphosphate reductase subunit beta	1.61	5.5E-19	00568 3,4-dihydroxy-2-butanone 4-phosphate synthase	7.35	2.08E-05	01757 hypothetical protein	2.15	4E-04
00653 Ribonucleoside-diphosphate reductase 1 subunit alpha	1.79	6.0E-19	00575 Bacterial transcription activator, effector binding domain	-9.06	9.72E-06	01606 hypothetical protein	-7.17	5E-07
00654 Adenylosuccinate lyase	1.90	9.5E-20	00596 Ribosome-associated factor Y	1.58	3.07E-04	01759 hypothetical protein	2.25	3E-07
00667 hypothetical protein	1.60	2.6E-16	00602 LPS-assembly protein LptD precursor	-1.58	1.37E-03	01442 Mu-like prophage I protein	3.22	2E-04
00695 hypothetical protein	1.99	1.8E-07	00612 Tyrosine recombinase XerC	2.61	9.46E-07	00513 putative transcriptional regulatory protein pdtaR	-5.35	2E-03
00706 EamA-like transporter family protein	-1.67	7.7E-09	00629 YGGT family protein	-1.66	4.01E-03	01747 Ankyrin repeats (3 copies)	1.85	8E-06
00715 hypothetical protein	-2.42	5.6E-12	00663 tRNA-Arg(tcg)	-12.27	1.27E-35	00633 EamA-like transporter family protein	-1.89	2E-04
00723 Modulator of drug activity B	-1.65	3.1E-06	00668 hypothetical protein	8.85	1.27E-04	00989 hypothetical protein	-5.69	6E-04
00933 hypothetical protein	-1.59	3.0E-15	00735 hypothetical protein	-6.37	4.99E-03	01656 hypothetical protein	2.03	7E-06
00936 Fumarate hydratase class II	1.88	9.6E-22	00769 hypothetical protein	-8.66	7.18E-05	01345 mRNA interferase MazF	-1.57	1E-04
00938 Blue-light-activated histidine kinase 2 (LOV-HK)	1.59	9.3E-06	00773 hypothetical protein	1.91	1.42E-06	00011 zinc-responsive transcriptional regulator	-1.90	1E-07
00941 PKHD-type hydroxylase	1.50	4.6E-05	00808 Anaerobic glycerol-3-phosphate dehydrogenase subunit C	1.69	9.36E-04	01429 hypothetical protein	2.75	6E-04
00946 Nickel uptake substrate-specific transmembrane region	1.65	2.4E-20	00816 hypothetical protein	-8.63	6.70E-05	00285 Outer membrane efflux protein	-1.85	1E-04
00987 Methionine aminopeptidase 1	1.55	8.1E-11	00823 hypothetical protein	3.64	9.45E-12	00286 Fatty acid metabolism regulator protein	-2.05	1E-06
00988 Translation initiation factor IF-1	1.85	3.1E-14	00832 N5-glutamine S-adenosyl-L-methionine-dependent methyltransferase	-7.89	2.23E-04	01082 hypothetical protein	1.88	8E-04
00989 Uracil DNA glycosylase superfamily protein	1.76	5.5E-19	00851 Dihaem cytochrome c	2.30	2.29E-03	00619 Putative monooxygenase YcnE	-3.94	2E-22
00995 Adenylate kinase	1.72	7.4E-20	00853 Dihaem cytochrome c	2.25	3.04E-03	00620 Modulator of drug activity B	-2.93	2E-15
01009 Nickel uptake substrate-specific transmembrane region	2.00	3.4E-13	00854 Cytochrome c-type protein SHP precursor	4.90	6.92E-13	00618 NADPH dehydrogenase	-2.96	1E-21
01055 fec operon regulator FecR	1.51	5.7E-03	00856 Phosphoethanolamine transferase EptA	2.18	2.23E-03	00284 Cobalt-zinc-cadmium resistance protein CzcB	-1.68	1E-03
01192 Uracil-DNA glycosylase	1.80	5.4E-11	00880 hypothetical protein	-1.52	4.99E-03	01734 hypothetical protein	2.41	3E-07
01201 hypothetical protein	2.04	1.0E-10	00885 Methylated-DNA--protein-cysteine methyltransferase, inducible	-8.94	1.53E-05	00617 EamA-like transporter family protein	-2.74	4E-17
01211 fec operon regulator FecR	1.79	4.2E-08	00949 Peptide methionine sulfoxide reductase MsrB	-8.38	1.04E-04	00425 Aerotaxis receptor	-1.96	5E-10
01212 RNA polymerase sigma factor YlaC	1.75	2.2E-10	00959 hypothetical protein	-1.56	1.65E-03	00991 hypothetical protein	-5.19	4E-03

01246 putative D,D-dipeptide transport ATP-binding protein DdpF	1.57	4.2E-14	01021 Paraquat-inducible protein A	-8.38	8.47E-05	00630 Sodium:sulfate symporter transmembrane region	-1.57	4E-04
01375 hypothetical protein	1.51	1.4E-15	01045 tRNA-Phe(gaa)	-1.87	1.65E-05	00259 hypothetical protein	3.15	4E-03
01420 CTP synthase	1.60	1.1E-17	01062 hypothetical protein	-6.73	2.84E-03	00631 General stress protein 14	-2.82	3E-11
01442 DNA topoisomerase 1	1.53	7.8E-19	01067 Ferric-pseudobactin 358 receptor precursor	-7.11	1.79E-03	01083 Ferric-pseudobactin 358 receptor precursor	2.53	2E-04
01698 Phosphoserine phosphatase	1.74	7.6E-21	01069 hypothetical protein	9.07	1.35E-32	00013 CDP-diacylglycerol--inositol 3-phosphatidyltransferase	-2.98	2E-20
01699 Transaldolase	1.61	5.5E-21	01070 Fic/DOC family protein	7.96	6.58E-06	00015 1-acyl-sn-glycerol-3-phosphate acyltransferase	-2.64	2E-12
01744 Acyl carrier protein	1.87	1.8E-23	01081 hypothetical protein	-7.57	5.39E-04	00012 hypothetical protein	-3.74	4E-14
01793 Spermidine/putrescine import ATP-binding protein PotA	2.17	1.1E-21	01083 LemA family protein	1.69	5.03E-04	01485 hypothetical protein	-6.54	3E-05
01794 Putative 2-aminoethylphosphonate transport system permease protein PhnV	1.98	1.6E-17	01098 Sensor protein KdpD	6.63	2.33E-03	00169 Cytochrome c-552 precursor	-2.60	4E-09
01913 hypothetical protein	-1.74	4.4E-07	01102 hypothetical protein	8.92	1.51E-03	00014 Phosphatidate cytidylyltransferase	-2.67	1E-14
01922 Ferrous iron transport protein B (*feoB*)	2.71	1.1E-23	01105 Phage integrase family protein	7.50	9.80E-06	00884 Fructose-1-phosphate phosphatase YqaB	2.27	3E-11
01954 hypothetical protein	2.41	1.3E-21	01205 bifunctional dihydroneopterin aldolase/dihydroneopterin triphosphate 2'-epimerase	-8.44	6.88E-05	00946 hypothetical protein	-2.50	5E-03
01955 Imelysin	1.76	4.6E-15	01216 2-aminoadipate transaminase	-2.87	2.54E-06	01443 hypothetical protein	2.61	6E-05
01956 Fatty acid hydroxylase superfamily protein	2.09	1.8E-12	01250 ATP-dependent RNA helicase RhlE	4.19	6.38E-20	00914 hypothetical protein	2.37	6E-11
01969 Sensor protein ZraS	1.51	1.9E-08	01263 ribonuclease H	-8.82	2.01E-05	00426 Methyl-accepting chemotaxis protein IV (*mcp4*)	-1.59	1E-05
01976 fec operon regulator FecR	1.53	4.8E-06	01273 fec operon regulator FecR	-9.14	1.40E-05	01596 Sensor protein BasS	2.73	5E-11
02001 Quinone-reactive Ni/Fe-hydrogenase small chain precursor	-2.02	6.1E-21	01293 putative Ni/Fe-hydrogenase B-type cytochrome subunit	-3.32	4.42E-03	01293 Putative beta-lactamase HcpC precursor	-1.76	1E-07
02073 protoporphyrinogen oxidase	-1.63	1.9E-10	01313 Methyl-accepting chemotaxis protein II	-1.90	4.13E-05	00568 Sensor protein RstB	-1.62	2E-04
02112 50S ribosomal protein L28	1.51	8.3E-14	01316 Hydrogenase isoenzymes formation protein HypC	-9.62	1.37E-06	02034 Tyrosine recombinase XerC	-1.80	2E-04
02231 hypothetical protein	1.51	5.7E-06	01332 Flagellar biosynthetic protein FlhB	-9.90	1.48E-07	01175 hypothetical protein	-1.61	2E-07
02242 MerT mercuric transport protein	-2.19	8.8E-04	01400 hypothetical protein	-6.53	5.50E-03	02288 Putative formate dehydrogenase	3.52	1E-14

02260 Cyclic di-GMP phosphodiesterase response regulator RpfG	1.66	2.5E-09	01401 hypothetical protein	-8.26	1.47E-04	01087 Phosphoethanolamine transferase EptA	3.68	1E-16
02261 hypothetical protein	1.65	2.2E-08	01403 hypothetical protein	-7.22	1.11E-03	01484 hypothetical protein	-1.62	2E-04
00276 Cation/acetate symporter ActP (*actP*)	2.41	7.4E-12	01407 hypothetical protein	-6.42	4.85E-03	01307 Sensor protein ZraS	-1.99	8E-05
00278 Cation/acetate symporter ActP (*actP*)	2.54	5.8E-11	01409 Virulence sensor protein BvgS precursor	-2.90	3.19E-03	01088 PAP2 superfamily protein	4.22	1E-14
00279 Inner membrane protein YjcH (*yjcH*)	1.56	4.0E-06	01412 hypothetical protein	-7.74	3.12E-04	01141 Lactate utilization protein C	1.64	5E-04
00056 Isochorismatase family protein	2.33	1.7E-11	01418 hypothetical protein	7.97	1.86E-04	01508 Transcriptional activator NphR	1.59	5E-05
00074 Fumarate reductase iron-sulfur subunit	1.51	5.2E-14	01441 IMPACT family member YigZ	-7.01	1.94E-03	01740 transport protein TonB (*tonB*)	1.55	2E-03
00077 NADH-quinone oxidoreductase subunit N	1.95	7.0E-21	01444 Methyl-accepting chemotaxis protein IV	2.83	8.01E-04	01008 Prophage CP4-57 integrase	-1.51	5E-05
00078 NADH-quinone oxidoreductase subunit M	1.80	1.6E-19	01456 Lipoteichoic acid synthase 1	-3.13	1.99E-03	01149 Membrane transport protein	-2.07	2E-03
00079 NADH-quinone oxidoreductase subunit L	1.96	1.3E-23	01467 Peptidoglycan O-acetyltransferase	6.78	1.02E-05	02315 Formate dehydrogenase iron-sulfur subunit	1.99	1E-05
00086 NADH-quinone oxidoreductase chain 1	1.54	4.9E-17	01497 von Willebrand factor type A domain protein	2.46	1.81E-03	01603 Prophage CP4-57 integrase	-2.48	8E-11
00106 hypothetical protein	2.28	1.5E-04	01500 Bifunctional adenosylcobalamin biosynthesis protein CobP	-7.90	2.41E-04	01609 hypothetical protein	-1.61	6E-04
00113 Putative multidrug export ATP-binding/permease protein	1.53	2.7E-02	01582 Flagellar filament 33 kDa core protein	2.83	2.14E-10	01817 CheW-like domain protein	-2.29	7E-04
00116 Regulatory protein PchR	3.38	4.3E-25	01588 Chaperone protein YajL	1.85	4.92E-04	02111 phosphoglycerol transferase I (*mdoB*)	1.77	1E-05
00118 Transcriptional repressor RcnR	-1.87	2.4E-13	01610 hypothetical protein	-8.06	1.56E-04	00290 Quinohemoprotein amine dehydrogenase subunit gamma	-6.10	1E-04
00122 Cytochrome c-552 precursor	-1.74	3.7E-14	01723 Flagellar motor switch protein FliM	1.72	2.79E-03	01507 Spermidine/putrescine import ATP-binding protein PotA	1.71	1E-04
00130 Periplasmic nitrate reductase, electron transfer subunit precursor	-1.93	4.8E-11	01724 Flagellar P-ring protein precursor	1.58	1.39E-04	01102 hypothetical protein	1.53	1E-05
00133 Periplasmic nitrate reductase precursor	-1.72	5.8E-13	01776 hypothetical protein	-1.54	2.56E-03	01398 Chaperone protein ClpB	-2.00	4E-06
00220 S-adenosylmethionine synthase	1.58	5.4E-17	01794 Methylisocitrate lyase	1.86	6.72E-05	02237 Regulatory protein PchR	-2.77	4E-12
00221 Acetyl-coenzyme A carboxylase carboxyl transferase subunit beta	1.62	2.0E-16	01795 2-methylcitrate synthase	2.06	1.25E-05	00170 Cytochrome c-type protein NrfH	-2.56	5E-12

00254 Fibrobacter succinogenes major domain (Fib_succ_major)	2.58	9.2E-18
00275 Putative nucleotidyltransferase substrate binding domain protein	2.35	7.1E-06
00308 hypothetical protein	1.61	5.7E-16
00348 hypothetical protein	1.70	2.0E-15
00350 YceI-like domain protein	2.58	1.1E-21
00358 ECF RNA polymerase sigma factor SigE	2.31	1.8E-12
00487 hypothetical protein	-1.58	4.0E-03
00492 hypothetical protein	-1.81	1.4E-15
00542 NADPH-dependent FMN reductase	-1.70	2.1E-13
00687 Cytochrome c-type protein SHP precursor	-1.89	1.2E-14
00806 Cytochrome c-type protein TorY	-1.82	2.4E-20
00925 Ankyrin repeats (3 copies)	2.08	1.5E-10
00939 Transcriptional regulatory protein WalR (*walR*)	2.60	1.5E-20
00948 hypothetical protein	1.80	8.3E-12
01056 Ferrichrome receptor FcuA precursor (*fcuA*)	1.75	2.3E-06
01193 Sulfoacetaldehyde dehydrogenase	2.07	1.6E-27
01194 Acetolactate synthase	2.31	1.7E-32
01325 Isocitrate dehydrogenase [NADP]	1.57	3.1E-15
01923 hypothetical protein	3.35	1.7E-16
01933 hypothetical protein	1.96	1.2E-11
01934 hypothetical protein	2.74	3.4E-18
01953 Imelysin	2.40	4.8E-30
02050 Helix-hairpin-helix motif protein	2.86	1.4E-36
02230 hypothetical protein	1.98	1.7E-10

01819 Transport protein TonB (*tonB*)	-1.95	1.91E-05
01820 Biopolymer transport protein ExbD (*exbD*)	-1.84	5.30E-05
01869 50S ribosomal protein L4	-1.53	6.04E-05
01879 50S ribosomal protein L24	-1.87	1.09E-03
01908 Citrate lyase subunit beta-like protein	1.61	1.47E-03
01914 Methyl-accepting chemotaxis protein IV	-7.72	4.22E-04
01917 HTH-type transcriptional repressor AseR	-10.74	1.27E-11
01919 putative permease	-1.71	4.84E-03
01979 NnrS protein	-6.93	2.08E-03
02006 Putative acetyltransferase EpsM	7.63	3.33E-03
02007 Putative pyridoxal phosphate-dependent aminotransferase EpsN	9.02	4.83E-06
02008 UDP-N-acetyl-alpha-D-glucosamine C6 dehydratase	7.85	4.28E-05
02012 Tyrosine-protein kinase etk	8.60	7.70E-08
02039 hypothetical protein	-8.78	2.10E-05
02045 hypothetical protein	2.40	1.96E-08
02055 Outer membrane lipoprotein Blc precursor	2.03	2.50E-07
02056 hypothetical protein	2.30	2.07E-06
02061 Transcriptional repressor MprA (*mprA*)	8.56	1.22E-05
02067 Urease accessory protein UreE	-8.09	1.42E-04
02068 Urease subunit alpha	-7.17	1.19E-03
02070 Urease accessory protein UreD	-6.63	3.40E-03
02079 Bifunctional NMN adenylyltransferase/Nudix hydrolase	-8.80	2.36E-05
02081 Response regulator PleD	-7.55	5.32E-04
02082 Transcriptional activator protein CopR	-6.83	2.48E-03
02083 Methyl-accepting chemotaxis protein IV	2.09	2.75E-04

01613 hypothetical protein	-2.26	6E-04
01607 hypothetical protein	-5.63	2E-03
01608 hypothetical protein	-1.88	2E-03
02148 AI-2 transport protein TqsA	-1.73	4E-03
01138 Glycolate permease GlcA	1.91	2E-06
00254 10 kDa chaperonin	-1.78	4E-09
02238 Ferrichrome receptor FcuA precursor (*fcuA*)	-2.68	2E-03
00918 Inner membrane protein YedI	1.63	1E-05
00331 Methyl-accepting chemotaxis protein 4	-1.91	2E-04

	02129 Spore protein SP21	1.60	9.84E-05	
	02141 Copper chaperone CopZ	-8.09	1.60E-04	
	02153 Cell wall-associated hydrolase	2.50	3.02E-06	
	02163 Methyl-accepting chemotaxis protein I	3.16	1.60E-10	

Supplementary Table 5. *A. butzleri* pyruvic acid and glucose related DEGs after 2 h of incubation in DMEM. The table shows logCPM, *p value* (< 0.05) and FDR (< 0.05) values of differentially expressed genes linked to pyruvate and glucose. The column "gene" shows the gene number and his locus tag. The logFC values indicated result higher than 1.5 or lower than 1.5 (except for LMG 11119 locus tag 01408 and 02015, logFC > 1.40).

gene	logFC	logCPM	PValue	FDR
Strain LMG 10828^T				
00081 Pyruvate kinase	-1.67	9.32	2.21E-12	7.53E-12
01697 UDP-glucose 6-dehydrogenase	1.64	7.11	4.45E-12	1.47E-11
02178 UTP--glucose-1-phosphate uridylyltransferase	1.97	9.28	3.70E-15	1.50E-14
02179 Glucose-6-phosphate isomerase	2.02	8.51	5.08E-16	2.18E-15
01698 dTDP-glucose 4,6-dehydratase	2.22	6.84	1.32E-17	6.40E-17
02286 Phosphoenolpyruvate carboxykinase [ATP]	2.31	7.97	3.98E-20	2.34E-19
01704 UDP-glucose 4-epimerase	2.31	6.68	9.20E-20	5.24E-19
02093 Glucose-1-phosphate thymidylyltransferase 2 (*rmlA*)	2.48	5.89	6.84E-20	3.93E-19
02095 dTDP-4-amino-4,6-dideoxy-D-glucose transaminase	2.62	5.41	1.37E-20	8.38E-20
02094 dTDP-glucose 4,6-dehydratase	2.77	5.76	2.43E-22	1.65E-21
Strain LMG 11119				
01408 Phosphoenolpyruvate-carboxykinase ATP	1.44	7.29	3.91E-15	6.44E-15
02015 Pyruvate dehydrogenase E1 component	1.45	9.01	1.14E-13	1.78E-13
00007 Glucose-1-dehydrogenase-2	1.52	2.65	6.30E-04	7.37E-04
00897 Glucose-1-phosphate-cytidylyltransferase	2.59	4.36	1.17E-16	2.02E-16
00908 UDP-glucose-4-epimerase	2.70	4.98	1.44E-24	3.36E-24
02067 UTP glucose-1-phosphate uridylyltransferase	2.78	7.88	5.39E-36	2.08E-35
02068 Glucose-6-phosphate isomerase	3.53	7.06	8.78E-45	5.25E-44
02176 Glucose-1-phosphate thymidylyltransferase 1 (*rmlA*)	1.66	7.52	7.86E-16	1.32E-15
02179 dTDP glucose 4,6 dehydratase	1.56	5.80	2.61E-14	4.17E-14
Strain 31				
01457 Glucose-1-phosphate thymidylyltransferase 2 (*rmlA*)	2.75	5.44	3.76E-17	2.44E-16
01458 dTDP-glucose 4,6-dehydratase	2.82	4.96	2.57E-16	1.51E-15
01459 dTDP-4-amino-4,6-dideoxy-D-glucose transaminase	2.65	3.31	3.71E-09	9.29E-09
01703 Phosphoenolpyruvate carboxykinase [ATP]	2.29	8.35	9.15E-14	3.7E-13
01809 UDP-glucose 4-epimerase	2.54	3.49	7.26E-09	1.78E-08
02010 dTDP-glucose 4,6-dehydratase	2.36	3.39	1.74E-08	4.1E-08
02041 UTP--glucose-1-phosphate uridylyltransferase	2.18	9.57	2.31E-10	6.62E-10
02042 Glucose-6-phosphate isomerase	2.48	8.76	1.46E-15	7.49E-15

Supplementary Table 6. Annotation statistics of the 3 *A. butzleri* strains. In the table are indicated the genome size, coverage ((read count * read length)/genome size), number of total genes, number of total CDS, tRNA and hypothetical proteins number.

Strain code	LMG 11119	LMG 10828^T	31
Genomes size (Mbp)	2.3	2.31	2.13
coverage (X)	195.32	205.69	486.87
GC content (%)	26.88	26.88	26.98
Number of contings	51	26	25
total genes	2278	2317	2164
CDS	2236	2271	2120
tRNA	41	45	43
hypothetical proteins	535	553	491

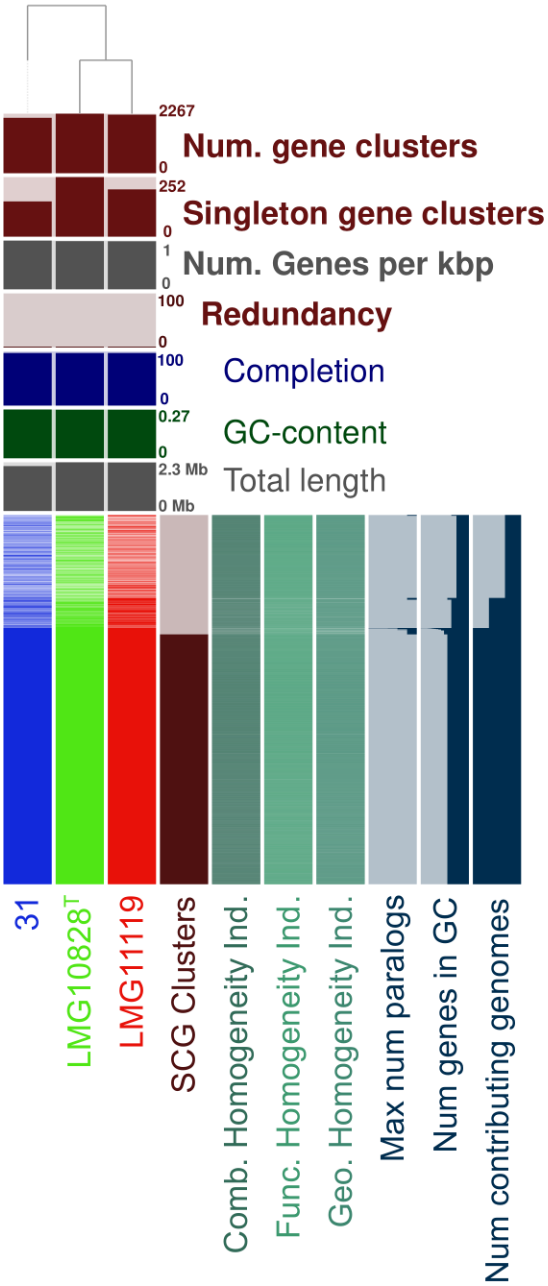

Supplementary figure 1. Genomic comparison between *A. butzleri* strains tested. The genomes of LMG 11119, LMG 10828^T (reference strain) and 31 have been compared with Anvi'o tool. The dendrogram shows gene cluster presence/absence, as well as general information about genomes.

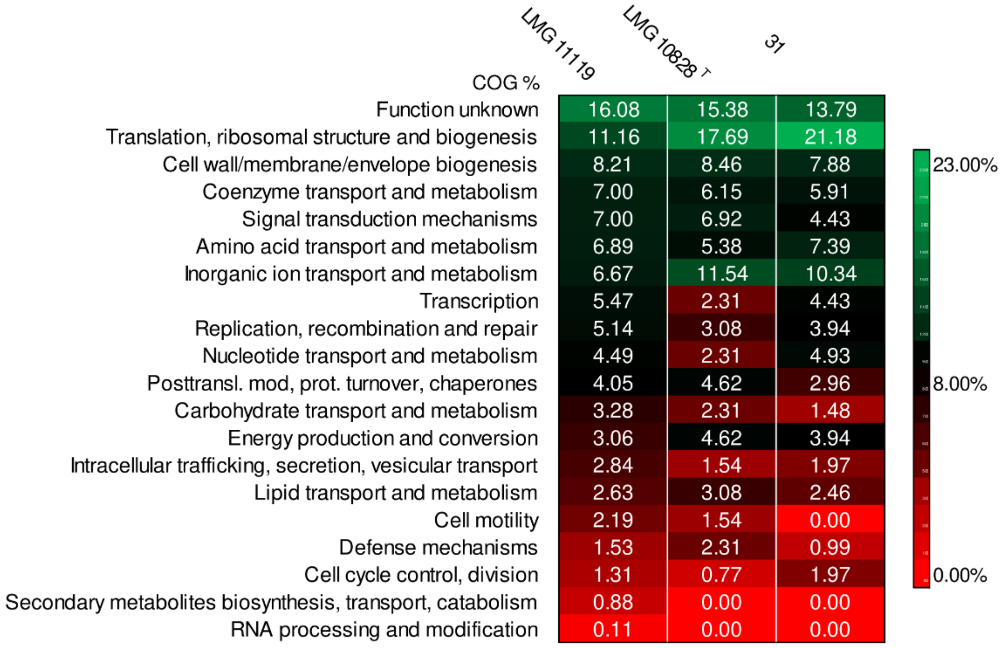

Supplementary figure 2. Gene classes of the DEGs after 2 h of *A. butzleri* incubation in DMEM. The heatmap shows percentages relating to COG gene classes of total DEGs detected after 2 h of *A. butzleri* incubation in DMEM.

COG %	LMG 11119 30'	LMG 10828T 30'	31 30'	LMG 11119 90'	LMG 10828T 90'	31 90'
Function unknown	15.87	25.64	19.15	14.86	25.00	21.77
Translation, ribosomal structure and biogenesis	3.17	0.00	2.13	7.43	0.00	9.52
Cell wall/membrane/envelope biogenesis	1.59	8.97	8.51	3.38	8.33	6.80
Coenzyme transport and metabolism	0.00	1.28	2.13	2.03	0.76	3.40
Signal transduction mechanisms	6.35	7.69	6.38	6.76	9.09	8.84
Amino acid transport and metabolism	6.35	3.85	2.13	6.76	4.55	2.72
Inorganic ion transport and metabolism	11.11	7.69	12.77	14.19	6.82	6.12
Transcription	12.70	5.13	2.13	4.73	6.06	5.44
Replication, recombination and repair	4.76	7.69	6.38	3.38	3.79	4.76
Nucleotide transport and metabolism	0.00	2.56	0.00	4.73	0.76	1.36
Posttransl. mod, prot. turnover, chaperones	6.35	2.56	0.00	2.03	4.55	5.44
Carbohydrate transport and metabolism	3.17	1.28	2.13	2.03	3.03	3.40
Energy production and conversion	9.52	15.38	12.77	16.89	15.91	7.48
Intracellular trafficking, secretion, vesicular transport	4.76	2.56	4.26	3.38	3.79	2.04
Lipid transport and metabolism	4.76	2.56	0.00	2.70	2.27	0.68
Cell motility	4.76	2.56	10.64	0.68	3.03	6.12
Defense mechanisms	1.59	2.56	4.26	1.35	2.27	1.36
Cell cycle control, division	1.59	0.00	0.00	0.68	0.00	1.36
Secondary metabolites biosynthesis, transport, catabolism	1.59	0.00	4.26	2.03	0.00	1.36
RNA processing and modification	0.00	0.00	0.00	0.00	0.00	0.00

26.00% 8.00% 0.00%

Supplementary figure 3. Gene classes of the DEGs after 30 minutes (30') and 90 minutes (90') of host-bacteria contact. The heatmap shows percentages relating to COG gene classes of total DEGs detected in virulence conditions at 30' and 90'.

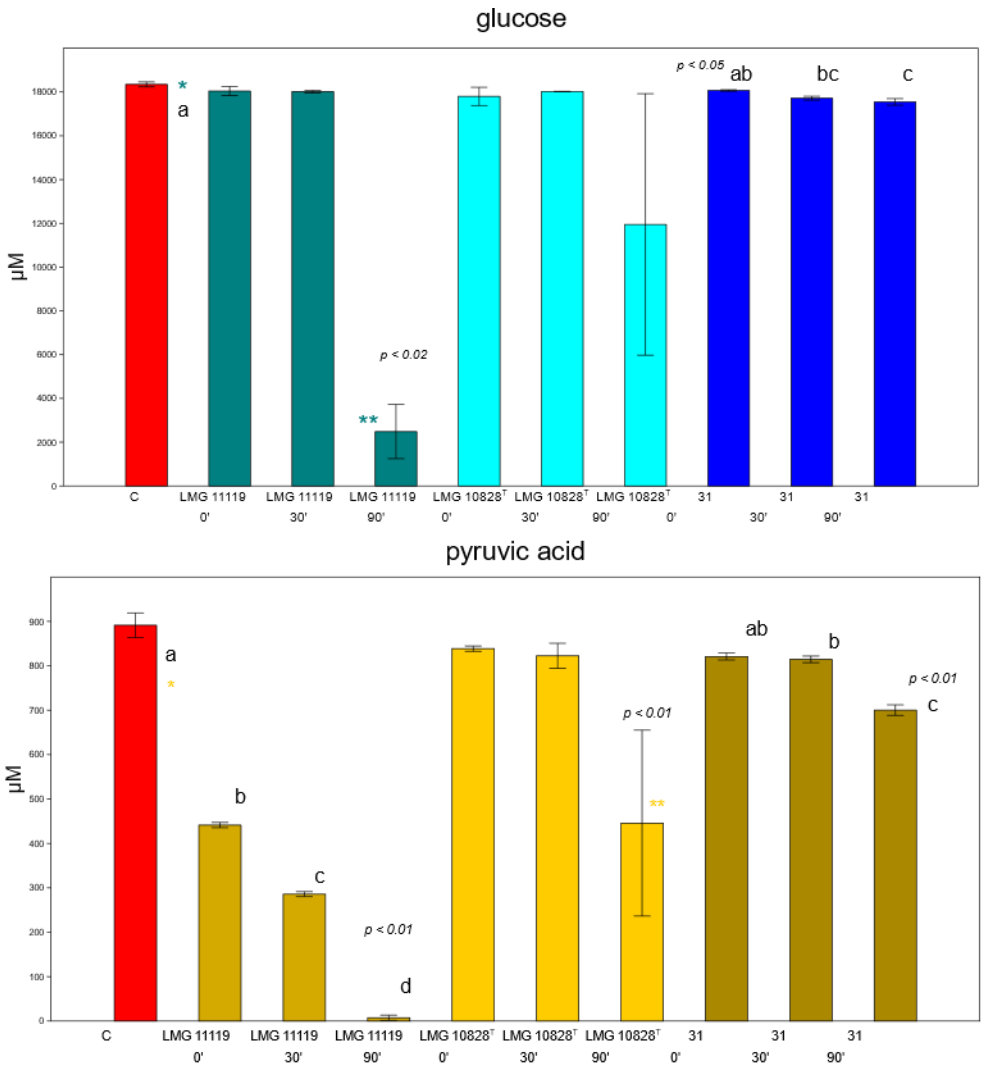

Supplementary figure 4. Glucose and pyruvic acid concentration of DMEM inoculated with *A. butzleri* strains (without human cells). The bar chart shows the concentrations in DMEM of glucose and pyruvic acid (µM). In the figure are indicated the strain codes and control (normal DMEM, C). The different sampling times are indicated as 0' (after acclimation), 30' and 90'. The error bars represent the standard errors (Past3). The figure shows statistical analysis *p*-value. The statistical differences between strains are indicated near bars.
